# Impact of pH-adjusted fluoride and stannous solutions on the protective properties on the pellicle layer in vitro and in situ

**DOI:** 10.1038/s41598-024-53732-7

**Published:** 2024-02-09

**Authors:** N. Johannes, S. Hertel, V. Stoffel, C. Hannig, S. Basche, V. Schmitt, J. Flemming, M. Hannig

**Affiliations:** 1 Clinic of Operative and Pediatric Dentistry, Medical Faculty Carl Gustav Carus, TU Dresden, Fetscherstraße 74, 01307 Dresden, Germany; 2 Clinic of Operative Dentistry, Periodontology and Preventive Dentistry, University Hospital, Saarland University, Building 73, 66421 Homburg, Saar Germany

**Keywords:** Fluoride, Stannous solutions, Pellicle, Erosion, Biofilm formation, Preclinical research, Dental caries

## Abstract

This study evaluates the ideal pH for anti-erosion and anti-adherent efficacy of fluoride and stannous solutions (sodium fluoride (SF), amine fluoride (AF), sodium monofluorophosphate (SMFP), stannous fluoride (SnF_2_) with 500 ppm fluoride concentration each and stannous chloride (SnCl_2_, 1563 ppm stannous)). In vitro, solutions were tested at pH 4.5 and 5.5. The main in situ experiments were carried out at the pH of 4.5: For pellicle formation 6 volunteers wore bovine enamel slabs intraorally for 1 min, rinsed with 8 ml solution for 1 min and continued for up to 30 min/8 h. Physiological pellicle samples served as controls. After incubation in HCl (2.0, 2.3) for 2 min mineral release was determined photometrically. Bacterial counts on 8 h biofilms were determined by fluorescence microscopy (BacLight™ and DAPI with Concanavalin A). Modification of the pellicle ultrastructure was examined by TEM. Statistical analysis was performed using Kruskal–Wallis and Mann–Whitney-U tests with Bonferroni-correction (*p* < 0.05). SnF_2_ showed a significant erosion protection. AF, SnF_2_, and SnCl_2_ were most anti-adherent. SnF_2_ and SnCl_2_ caused a pronounced basal pellicle with stannous precipitates. Compared to other fluoride monosubstances, stannous ions offer greater protection against erosive acidic attacks. Stannous ions act as crucial co-factor in this process.

## Introduction

The prevention of caries and erosion is essentially based on the daily topical application of fluoride-containing oral care products. Fluoride compounds are considered as essential in toothpastes and mouthwashes as they reduce the incidence of caries and, to a limited extent, erosion^1–6^. Investigation of the underlying bioactive interactions at the tooth surface, in particular with the ubiquitous pellicle as a physiological protein layer, is essential for a better understanding of the exact mechanisms of action^7^. The pellicle is formed by selective adsorption of salivary proteins to the tooth surface and acts as a protective semi-permeable barrier against acid-induced demineralization^7,8^. Besides, the pellicle has receptors that facilitate bacterial colonisation of the dental hard tissues^8,9^. A number of in vitro and in situ studies have investigated the possibility of modifying the pellicle layer to enhance its erosion-preventive and anti-adhesive properties by topical application of fluoridated rinses or toothpastes^3,10–14^.

It has been shown that the metal cations of the fluorides significantly influence their anti-adherent and anti-erosive efficacy^15–17^. In particular, stannous ions in fluoride compounds such as stannous fluoride, but also in fluoride-free stannous solutions such as stannous chloride, significantly improved erosion- and caries protection of the in situ pellicle^18,19^. Stannous ions have been described to affect bacterial metabolism by inhibiting enzymes that metabolise sugars, leading to bacterial cell death and consequently reduced acidogenicity of the bacterial biofilm^20,21^. The effect of stannous fluoride on bacterial colonisation has not been extensively studied in situ. Two previously published studies examined the effect of SnF_2_ and NaF on enamel specimens by electron microscopy^11,22^. However, the authors noted that there was limited assessment of the results, as both previous studies abstained from capturing TEM images and solely acquired SEM images after being treated with SnF_2_^11,22^.

There is evidence that, in addition to the cation of the fluoride compound, the pH of the solution also affects the efficacy, particularly concerning the erosion protective potential^15,23,24^. Wiegand et al.^15^ used profilometry to investigate the erosion protective effect of different fluoride and stannous solutions at uniform fluoride concentrations and pH values in vitro. Profilometry does not account for demineralized enamel areas that have not been softened yet. Additionally, profilometric changes can only be detected at a depth of 1 µm or greater^25^. Nevertheless, the fluoride concentrations of the test solutions with 1000 ppm and 5000 ppm were significantly higher than commercial rinsing solutions^15^. The pH values of the solutions were adjusted to 3.9 and 7.0. Only the acidic amine and stannous fluoride solutions provided significant protection against erosion. The pH of the fluoride solution had a more pronounced effect on erosion protection than the fluoride concentration or the corresponding cation^15^. Another in vitro study on the erosion protective properties of highly concentrated sodium and stannous fluoride solutions showed, by atomic absorption spectroscopy and SEM, that the acidic stannous fluoride solution (pH 3.0) with 4000 ppm fluoride content provides significantly better erosion protective effects than the neutral (pH 7.0) sodium fluoride solution with a fluoride content of 20 000 ppm^23^. As the concentration of the solutions used in the study was not uniform, no statement can be made about the mode of action. There is some evidence for the effect of pH on erosion inhibition, but little for the effect on bacterial adherence. In the study by Kirsch et al.^26^, fluoride monosubstances in an acidic environment between 3.5 and 4.5 were particularly effective against bacterial biofilm formation and could therefore provide caries protection. However, effective fluoride deposition at the tooth surface in a pH dependant manner is also relevant for the caries preventive properties. Furthermore, the pH of the fluoride preparation has an impact on the interaction with the bacterial biofilm and antiadherent properties^2^.

To gain a better understanding of the basic interactions of fluoride compounds and stannous ions on the tooth surface, our scientific working group tested monosubstances such as sodium fluoride (SF), sodium monofluorophosphate (SMFP), amine fluoride (AF) and stannous fluoride (SnF_2_), but also stannous chloride (SnCl_2_) at a uniform fluoride concentration of 500 ppm in situ^24,26^. The stannous ion content was adjusted to the stannous ion content of a 500 ppm fluoride rinse (1563 ppm stannous ions). With regard to erosion protection SnF_2_ but also SnCl_2_ were found to be very effective in preventing acid-induced enamel defects, since they inherently have a very low pH (pH ≤ 4.5)^24^. However, this low pH could potentially initiate demineralisation on the enamel surface. A subsequent in situ study examining the impact of these solutions on the initial bacterial colonization and ultrastructural visualization through TEM also revealed the most anti-adherent effects for SnF_2_ and SnCl_2_^26^. Yet, the formation of subsurface pellicle in the enamel lacunae caused by the acidic environment of SnCl_2_ was also observed^26^.

In order to investigate whether a solution provides better protection against erosion or bacterial colonisation due to the acidic environment, it would be necessary to adjust the pH value of the solutions uniformly. It is assumed that acidic fluoride and stannous solutions have a stronger preventive effect than neutral ones, but an in situ experiment adjusting the pH of different fluoride and/or stannous solutions has not yet been carried out. Therefore, the aim of the present study was to investigate the effects of the pH of different fluoride and stannous monosubstances at the same fluoride concentration (500 ppm) on the erosion-preventing properties of pellicles in a preliminary in vitro test. The effects of the pH-adjusted test solutions (pH 4.5) on the erosion-preventing properties, pellicle ultrastructure and initial bacterial colonisation of the tooth surface will then be evaluated in situ.

## Material und methods

### Experimental setup

**Figure 1:**
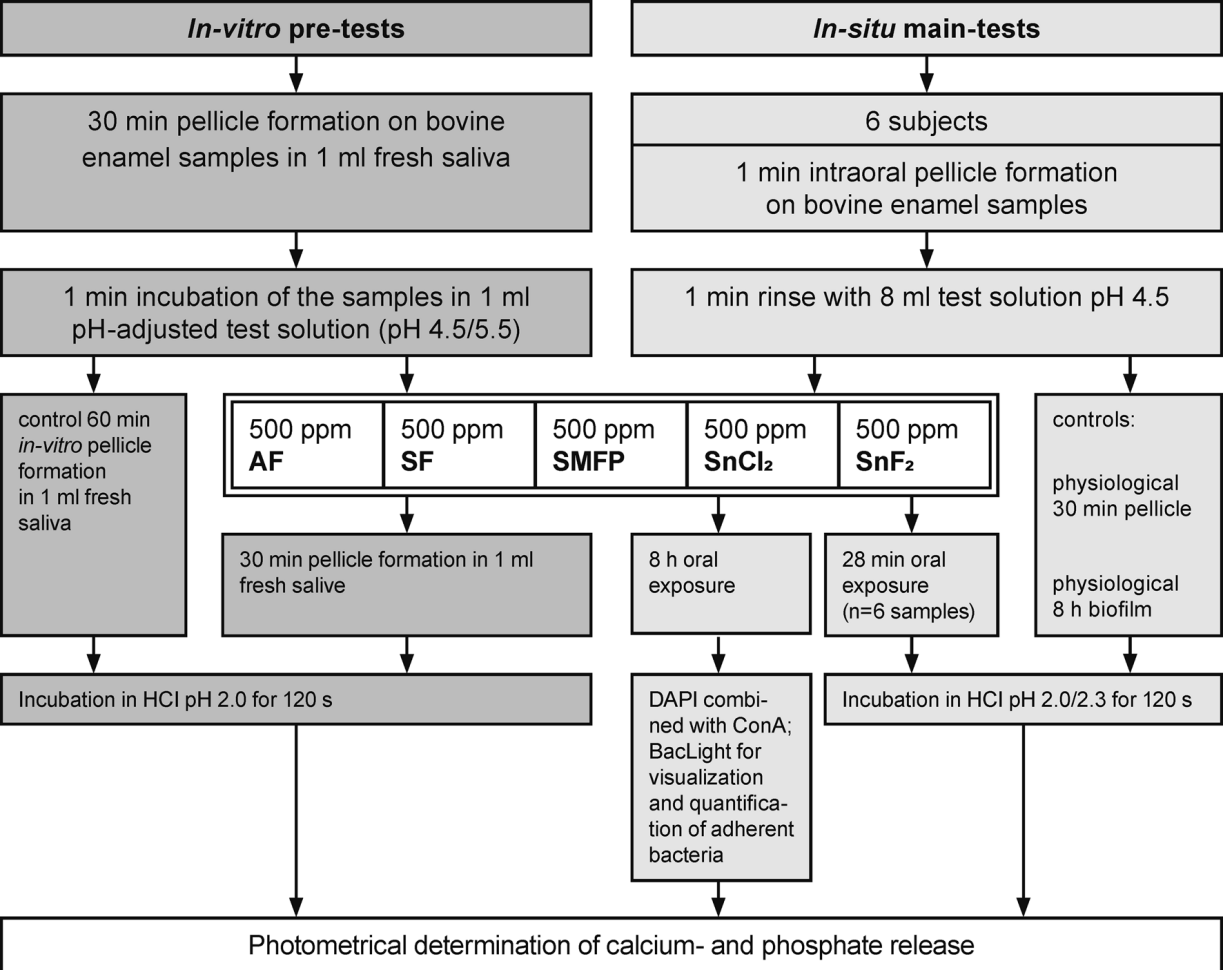
Flowchart depicting the sequence of erosion experiments.

In order to investigate the erosion protective properties of the fluoride and stannous test solutions at different pH values, preliminary tests were carried out in vitro at pH 4.5 and 5.5, which is shown in the supplementary. The main in situ studies were carried out at the pH of 4.5 (Fig. 1).

### Specimen`s preparation

Bovine enamel specimens (5 mm diameter, 19.63 mm^2^ surface area) were used for both the in vitro preliminary and in situ studies^24,27^. They were gained from the labial surfaces of bovine incisors of BSE-negative cattles (Holstein Friesian), all from the same abattoir. The adoption of bovine teeth was checked and approved by the Landesdirektion Sachsen (Regional Directorate of Saxony). The test specimens were sealed on all sides except the outer enamel surface with Optibond FL according to the manufacturer’s instructions. Exposed enamel surfaces were wet ground and polished up to 4000 grit. The resulting smear layer was removed by ultrasonication with 3% NaOCl for 3 min, followed by disinfection with 70% ethanol and distilled water. To ensure uniformity and standardisation, samples with an elliptical shape or visible enamel cracks were excluded. The remaining specimens without signs of structural enamel alterations were stored up to 24 h at 4 °C for experimental use^24,27^. The whole sealing and grinding procedure as well as the preincubation and cleaning of the samples was carried in a highly standardized manner as published previously^23,24^^.^

### Subjects

Six volunteers (20–48 years of age) agreed to participate in both in situ studies. They were caries-free, healthy non-smokers without signs of periodontitis and exhibited physiological salivary flow^24,27^. Prior the begin of the study, all volunteers had given their informed, written consent. The oral examination and the following study were performed at the Clinic of Operative Dentistry at the TU Dresden in Germany. The approval for the experimental design was granted by the Ethics Committee of the TU Dresden (EK 147,052,013), and all methods were carried out in accordance with the relevant guidelines.

For oral exposure of the test specimens, individual maxillary splints were fabricated for each subject, consisting of polymethyl methacrylate and stainless steel with buccal bars for up to 3 test specimens per side^13,26,27^. The specimens were fixed with polyvinyl siloxane impression material (Provil novo light, Kulzer GmbH, Hanau, Germany). For the erosion studies, each participant wore 6 slabs per test solution (3 slabs for HCl pH 2.0 and 3 slabs for HCl pH 2.3). The bacterial studies were performed with 4 slabs per participant per test solution.

### Fluoride and/or stannous monosubstance rinsing solutions

Five different pH- and concentration-adjusted fluoride and/or stannous monosubstances were used as test solutions. Monosubstances of sodium monofluorophosphate (SMFP) (Omya Schweiz AG, Oftringen, Switzerland), sodium fluoride (SF) (Ferdinand Kreutzer Sabamühle GmbH, Nürnberg, Germany), amine fluoride (AF) (Permcos GmbH, Arisdorf, Switzerland), stannous fluoride (SnF_2_) (Honeywell Speciality GmbH, Seelze, Germany) and stannous chloride (SnCl_2_) (Honeywell Speciality GmbH, Seelze, Germany) were prepared by dissolving the substances in distilled water to obtain a fluoride concentration of 500 ppm and/or a stannous concentration of 1563 ppm)^24^. The preparation is explained in Table 1. For the preliminary in vitro tests, the solutions were adjusted to pH 4.5 and 5.5 using NaOH and HCl.

**Table 1:** Preparation of 500 ppm fluoride solutions and 1563 ppm stannous solutions for dissolution in distilled water.

Monosubstance	molar mass (g/mol)	Fluoride molar mass	Weight/10 ml for 500 ppm fluoride (mg)
Sodium fluoride (SF)	41,99	18,9984 g/mol	11
Sodium monofluorphosphate (SMFP)	143,95	18,9984 g/mol	38
Stannous fluoride (SnF_2_)	156,69	2* 18,9984 g/mol	21
Amine fluoride (AF)	498,78	2* 18,9984 g/mol	199
Stannous chloride (SnCl_2_)	225,65	stannous analogue stannous in SnF_2_	30

### Pellicle formation in vitro

The experimental protocol for pellicle formation in vitro is attached in the supplementary.

For in vitro pellicle formation, samples were incubated in freshly collected saliva at 37 °C under gentle agitation for 60 min. After 30 min, the in vitro pellicle was modified with the MS (pH 4.5 or 5.5) for 1 min. As an in vitro control group, test specimens were incubated in saliva for 60 min without any rinsing.

### Pellicle formation in situ

For the in situ experiments, participants were instructed to brush their teeth with water 1 h before the test phase. They were also allowed to drink water during this time^24^. The subjects wore their maxillary splint with 6 enamel samples for 30 min. After 1 min of initial pellicle formation, they rinsed with the pH-adjusted test solution for 60 s. The splint was then left in the mouth for another 28 min to allow further pellicle maturation. For the control group, the participants wore their splints for 30 min without conducting any rinsing procedure^24,27^. The specimens were stored at 4 °C for a maximum of 30 min until analysis.

### Ex-vivo erosion and photometric determination of calcium and phosphate release

To assess the erosion protective properties of the respective solutions, the specimens were embedded in silicone impression material at the bottom of a 2 ml Eppendorf tube. A volume of 1000 µl HCl (pH of 2.0 or 2.3) was then added to each of two specimens. During the 120 s incubation period, regular lifting strokes were performed with a 100 µl pipette to ensure constant acid application. Every 15 s, a volume of 100 µl of the acid was removed for photometric analysis and replaced with 100 µl of fresh acid. Mineral loss was determined by measuring calcium and phosphate release into solution^28,29^. Therefore, photometrical assays based on the arsenazo III method (Calcium AS FS, DiaSys Diagnostic Systems GmbH, Holzheim, Germany) and the malachite green assay was applied. By binding to calcium, arsenazo III forms a bluish-violet complex in an acidic environment^28^. Malachite green forms a colour complex with phosphate^28–30^. Specifically, a volume of 10 µl of each sample was added with 100 µl of arsenazo III reagent for calcium determination and 200 µl of malachite reagent for phosphate determination. After 15 min, the absorbance at λ = 650 nm was recorded according to the standard curves. This procedure was always carried out in triplicate. The calcium and phosphate release was based on the mean photometric absorbance values for the samples and their surface area (19.63 mm^2^).

### Initial biofilm formation

Figure 1 shows the experimental setup for evaluating biofilm formation. The 6 volunteers were instructed to wear their splints for 8 h overnight. Prior, they were not allowed to consume anything except water and had to brush their teeth with water. For initial pellicle formation, the participants carried their splint with 2 specimens on each side for 1 min. Thereafter, they rinsed with the test solution at pH 4.5. For further biofilm formation, they continued to wear their splints for another 7 h and 58 min. As a control, the participants wore the splint for a period of 8 h without rinsing with any solution^26^. Samples were stored at 4 °C for a maximum of 30 min until the microscopic analysis.

### Visualization of bacterial adhesion and glucan formation

The blue-fluorescent dye 4',6-diamidino-2'-phenylindole (DAPI) penetrates both bacteria with intact membranes and those with damaged cell membranes^9,31,32^. Hence, it is not possible to distinguish between vital and avital bacteria. To visualize the interactions between bacteria and their surrounding glucan structures, simultaneous staining with DAPI and Concanavalin A (ConA) was performed. This staining method is able to represent glucan structures under fluorescence microscopy^32^. ConA specifically binds to α-mannopyranosyl and α-glucopyranosyl residues of glucans^32^. To prepare the working solution for DAPI and ConA staining, 245 µl of buffer, 5 µl of the ConA stock solution and 0.75 µl of the DAPI stock solution were mixed together and incubated with the enamel specimens in total darkness for 15 min. After rinsing the samples with saline solution, they were evaluated under the fluorescence microscope.

#### Bacterial viability assay (BacLight™)

The LIVE/DEAD® BacLight™ Bacterial Viability Kit (Invitrogen, Molecular probes, Darmstadt, Germany) is used for fluorescence-based vital staining of bacteria, enabling differentiation between vital and death bacteria based on their membrane integrity^33,34^. The green-fluorescent dye SYTO® 9 (component A, 1.67 mM/propidium iodide, 1.67 mM, 300 µL DMSO) can penetrate both bacteria with intact and bacteria with damaged membranes when used alone^35^. The additional red-fluorescent dye Propidium iodide (component B, 1.67 mM/propidium iodide, 18.3 mM, 300 µL DMSO) can only penetrate damaged membranes and displaces the SYTO® 9 dye when used in combination^35^. To stain the specimens, 2 µl of the stock solution consisting of equal parts of component A and component B was combined with 1 ml of saline solution. After a 10 min incubation in the resulting solution, the test specimens were rinsed with saline solution and subjected to evaluation under the fluorescence microscope.

#### Quantification of adherent bacteria using BacLight™ and DAPI and ConA

After analyzing the entire sample surface, 10 representative areas were selected and photographed for bacterial count and glucan score. Each selected area was captured twice and superimposed via the Axio Vision program to visualize blue-fluorescent bacteria and red-fluorescent glucan structures. The selected area size corresponded to 0.0156 mm^2^, which allows calculation of the number of bacteria per square centimetre accurately. Simultaneously, the stained glucan structures underwent assessment using a glucan score of 0–4^24^ (Table 2).

**Table 2:** Evaluation scheme for the extent of glucan formation of fluorescence microscopic analyses (glucan score).

Glucan score	Evaluation
0	No recognisable glucan rings
1	Individual glucan rings or bundles
2	Some distinct rings are visible, while there are also less prominent rings and clusters around the bacteria
3	At least 50% of bacteria have distinct glucan rings
4	Clear glucan rings in (almost) all bacteria

#### Ultrastructural analysis with Transmission electron microscopy (TEM)

TEM was used to visualise the ultrastructure of the pellicle in selected enamel samples from one subject. After an oral exposure of 8 h, the samples were fixed using a glutaraldehyde solution (2.5% glutaraldehyde, 1.5% formaldehyde in phosphate buffer). After osmication with 1% osmium tetroxide stock solution and 0.1 M cacodylate buffer, the enamel samples were dehydrated in an ascending ethanol series diluted in distilled water (50%, 70%, 90%, 100%), 100% acetone and an Araldite-acetone mixture (1:1), followed by embedding in an Araldite mixture (Plano, Wetzlar, Germany). Remaining dentin on the samples was removed using 1200-grit sandpaper. The enamel samples were decalcified in 1 M HCl and counter-embedded in an Araldite mixture. Ultrathin sections were made using Reichert Ultracut E (FEI Eindhoven, The Netherlands). If necessary, the samples were post-stained with uranyl acetate and lead citrate. TEM investigations were conducted using TEM TECNAI 12 Biotwin (FEI Eindhoven, The Netherlands).

#### Statistics

Statistical analysis of all experiments was performed using SPSS 28.0 (IBM, Ehningen, Germany). Calcium and phosphate release data after 120 s of incubation were analysed using the Kruskal–Wallis test, as the values were not normally distributed, followed by the Bonferroni correction. Fluorescence microscopic data were analysed using both the Kruskal–Wallis and Mann–Whitney-U tests, also followed by Bonferroni correction. The hypothesis was if the distribution of values is identical across the categories of groups. The significance level was set at *p* < 0.05 after Bonferroni correction. For the in situ erosion study, a post hoc power analysis was performed using G.POWER 3.1 (Heinrich-Heine-Universität, Düsseldorf, Germay). If the power is greater than 0.8, the result of the statistical test is considered reliable (Table 3).

**Table 3:** post hoc power analysis using G.Power 3.1 (Heinrich-Heine-Universität Düsseldorf, Düsseldorf, Germany). If the power is greater than 0.8, the result of the statistical test is considered reliable.

Erosion test	Power of the statistical analysis
Calcium release after incubation in HCl of pH 2.0	1.00
Calcium release after incubation in HCl of pH 2.3	1.00
Phosphate release after incubation in HCl of pH 2.0	0.99
Phosphate release after incubation in HCl of pH 2.3	0.99

## Results

### Calcium- and phosphate release in vitro

The results of the in vitro pre-tests are shown in the supplementary Fig. 1s. In summary, all fluoride and/or stannous-containing test solutions, but especially SnF_2_, lead to a reduction in calcium and phosphate release compared to the control. In addition, the monosubstances tended to show stronger effects at pH 4.5 (Fig. S1a) than at pH 5.5 (Fig. S1b).

### Calcium- and phosphate release in situ

**Figure 2:**
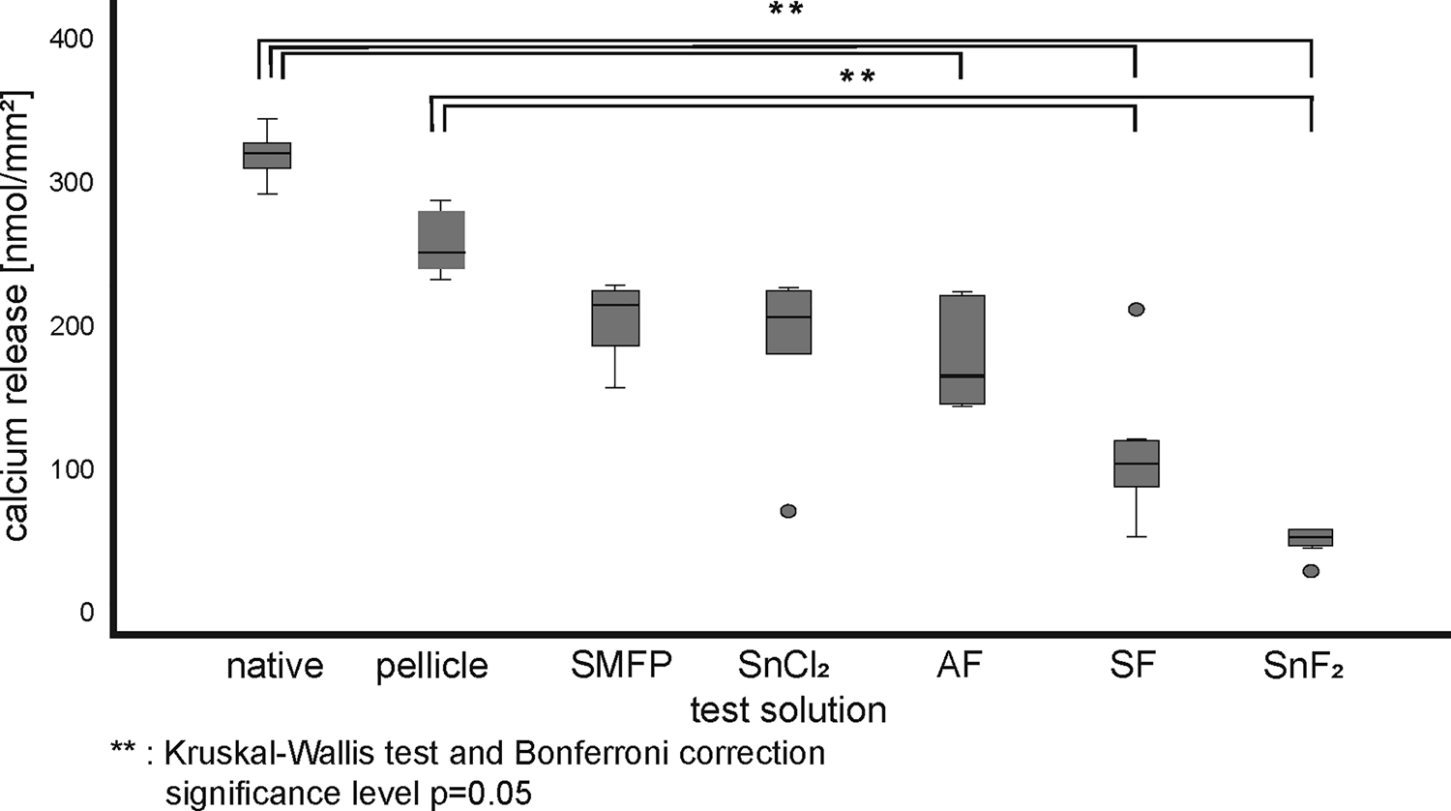
Calcium release from enamel specimens after 120 s of incubation in HCl at pH 2.0 following 30 min of in situ pellicle formation using rinsing substances at pH 4.5 for 1 min. Statistical analysis performed using the Kruskal–Wallis test and Bonferroni correction (*p* < 0.05).

The results of phosphate release from the specimens in situ is shown in the supplementary (Fig. S2, S4, S6 and S8). The results of calcium release from the in situ test specimens after incubation in HCl at pH 2.0 are shown in Fig. 2. Supplementary figure S3 shows the calcium release after 120 s incubation in HCl pH 2.3. Mineral release over the course of 2 min is shown in the supplementary (Fig. S5–S8). The mineral release increases almost linearly over the course of 2 min. The in situ pellicle reduces calcium and phosphate release compared to the native control group, although not significantly. When adjusting all test solutions to pH 4.5, there is a significant reduction in mineral release compared to the native control. Among all solutions adjusted to pH 4.5, only SnF_2_, consistently exhibits a significant improvement in the erosion-protective properties compared to the 30-min in situ pellicle (reduction of calcium release: − 79% at pH 2.0, *p* < 0.002; − 94% at pH 2.3, *p* < 0.001). The SnCl_2_ solution shows no significant improvement of the pellicle's erosion protection, similar to conventional fluoride solutions containing 500 ppm fluoride. Overall, the erosion protection of the solutions applied in situ is more pronounced than in the in vitro test series (average reduction of calcium release in vitro: − 45%, average reduction in situ: − 54%).

### Initial bacterial colonization

**Figure 3:**
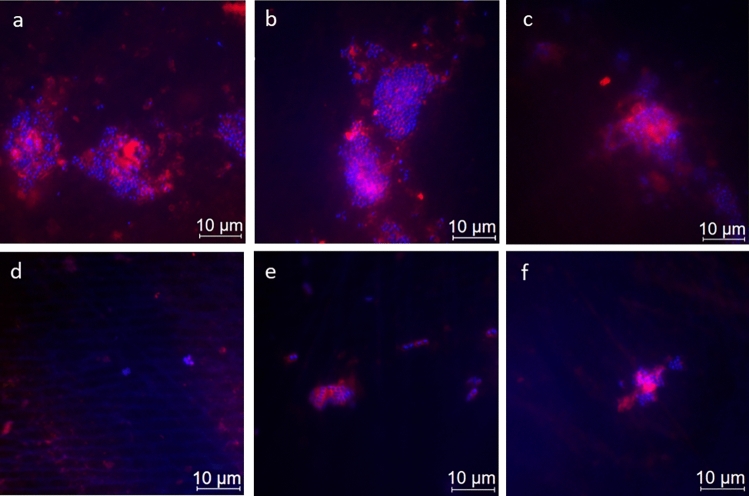
Representative fluorescence microscopic images showing combined DAPI and ConA staining after 8 h of in situ pellicle formation. DAPI (blue) highlights the number of bacterial cells adhering to the enamel surface, while ConA (violet) labels bacterial glucan agglomerates. The control group (**a**) shows the highest number of bacteria, similar to SF rinsing (**b**) and SMFP treatment (**c**) with sporadic glucan rings and diffuse extracellular polysaccharides around almost all adherent bacteria. SnF_2_ (**d**), SnCl_2_ (**e**), and AF (**f**) show reduced bacterial presence with less glucan rings and extracellular polysaccharides.

**Figure 4:**
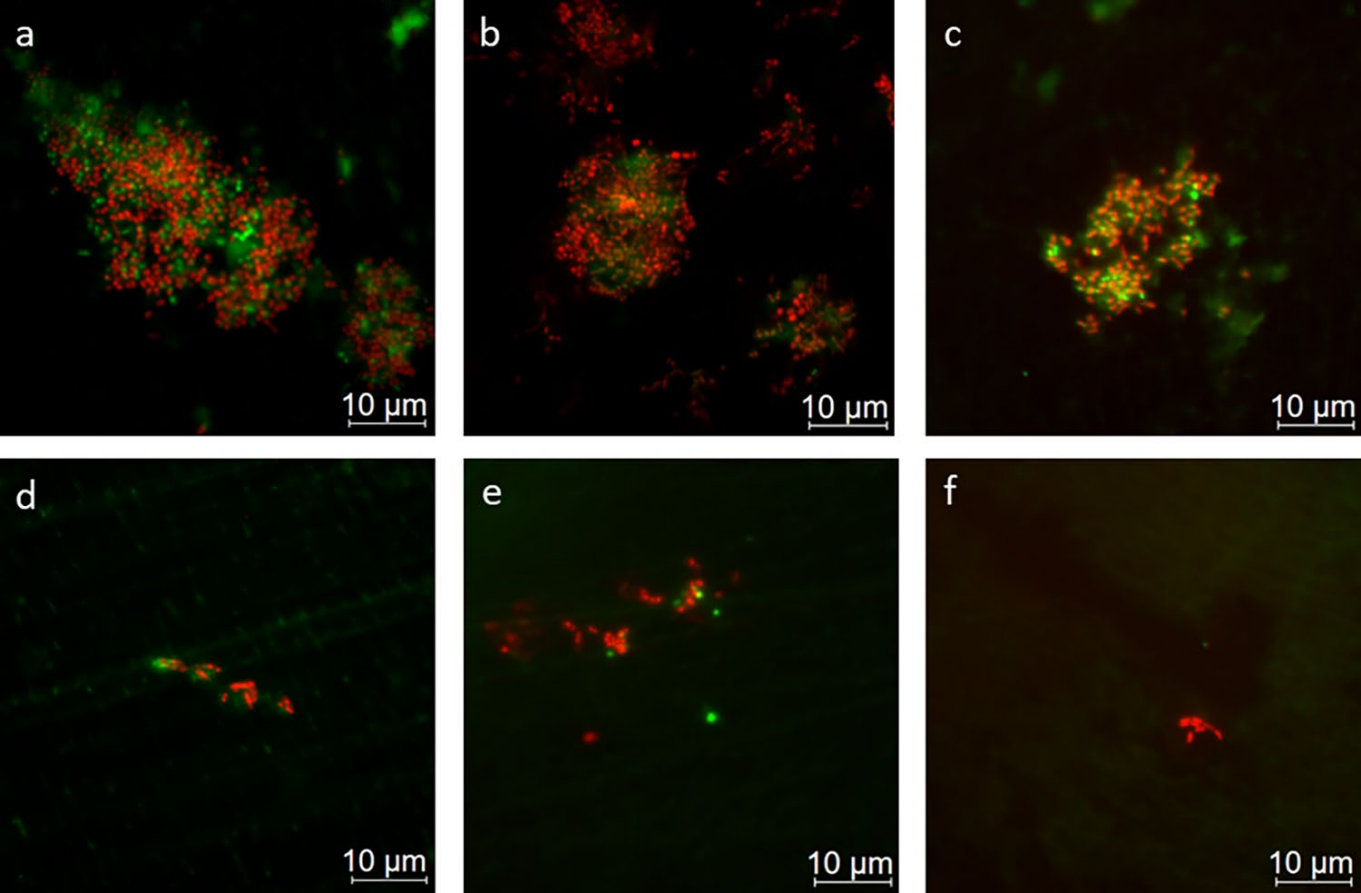
Representative fluorescence microscopy images illustrating the BacLight™ LIVE/DEATH staining. Vital (green) and avital (red) bacteria are observed after 8 h of in situ pellicle formation. The control group (**a**) shows the highest number of bacteria, similar to the groups treated with SF (**b**), SMFP (**c**) for 1 min each with a dense, multi-layered film of bacteria. SnF_2_ (**d**), SnCl_2_ (**e**), and AF (**f**) show reduced bacterial presence with isolated coccoid stacks.

**Figure 5:**
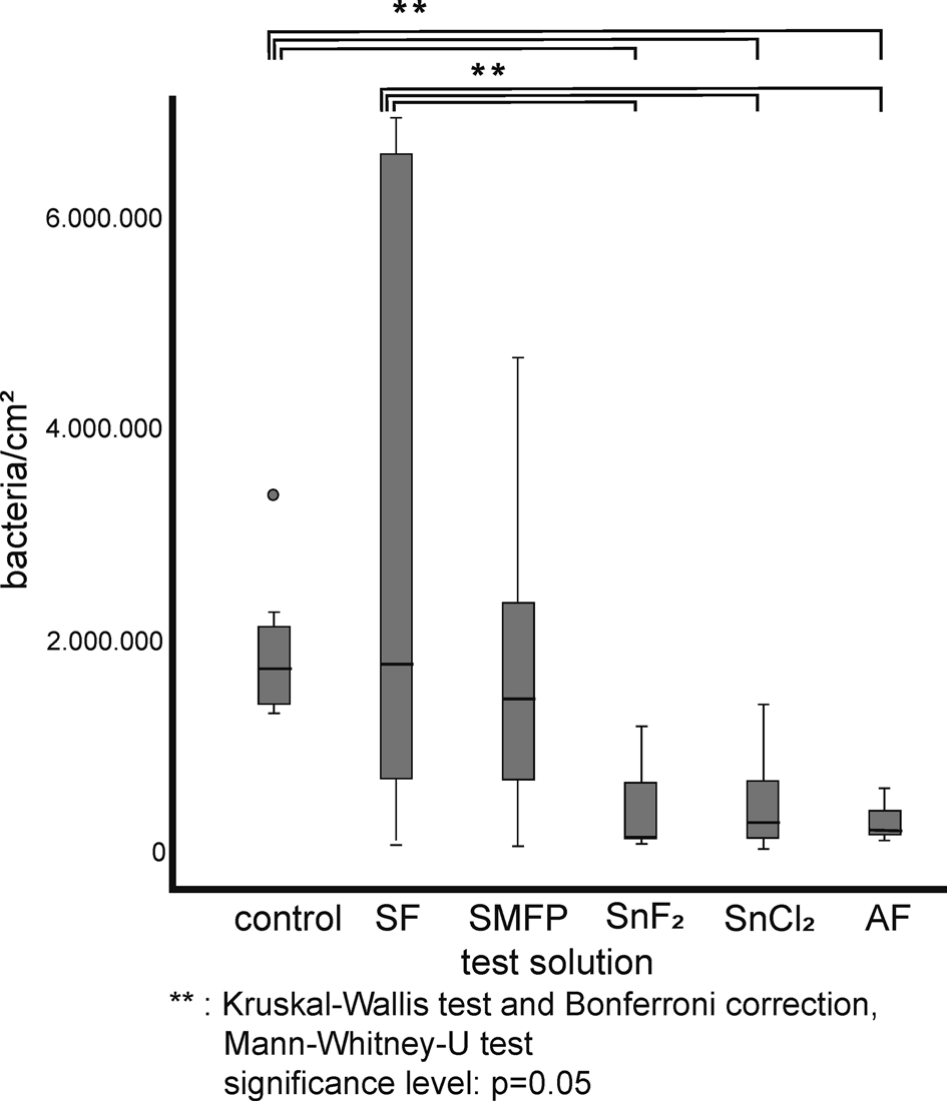
Quantification of adherent bacteria using DAPI staining presented in a box plot diagram. The number of adherent bacteria was assessed after 8 h of biofilm formation and 1-min rinsing with the monosubstances at pH 4.5. The bars represent significant differences, as determined by Kruskal–Wallis and Mann–Whitney-U tests.

**Figure 6:**
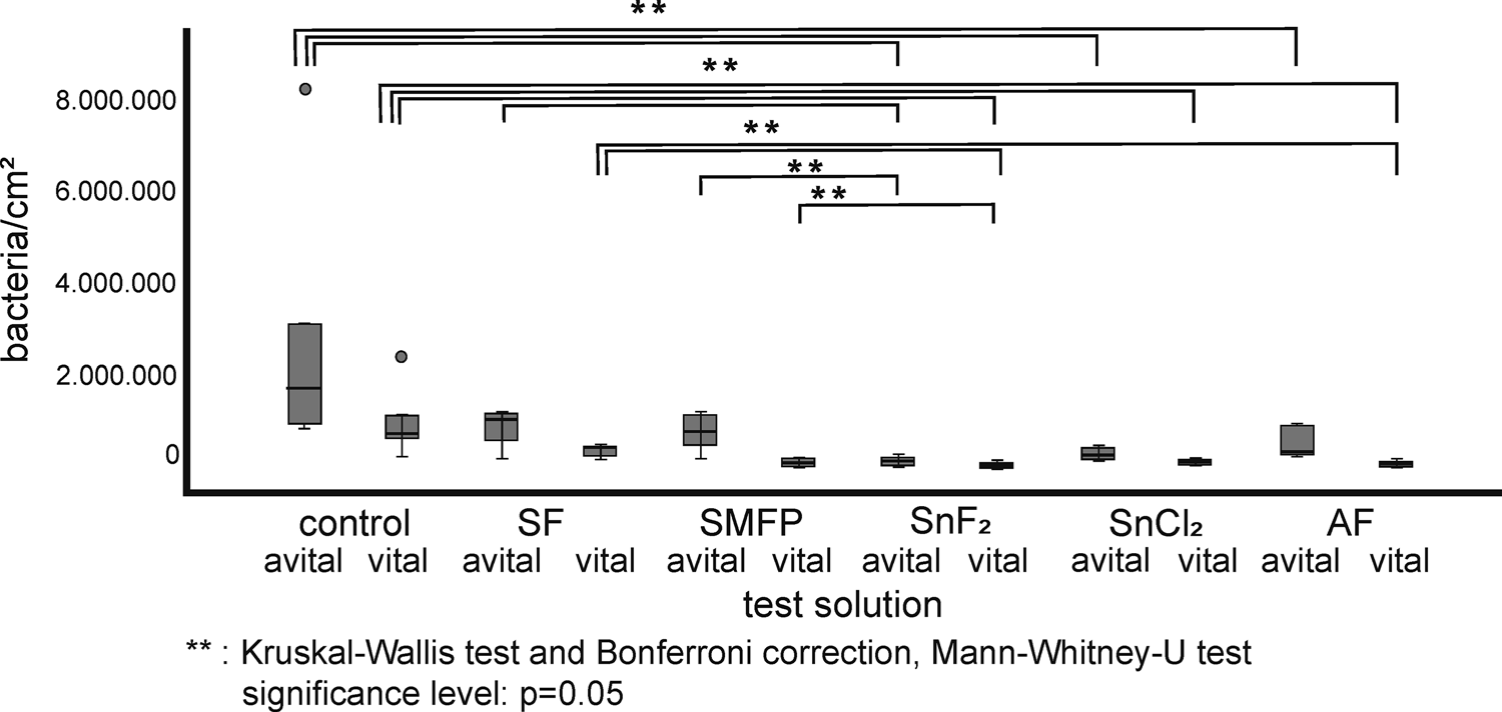
Quantification of vital and avital adherent bacteria using BacLight™ staining shown in a box plot diagram. The number of adherent bacteria was assessed after 8 h of biofilm formation and 1-min rinsing with the monosubstance test solutions at pH 4.5. Boxes with the same letter indicate significant differences, as determined by Kruskal–Wallis and Mann–Whitney-U tests.

**Figure 7:**
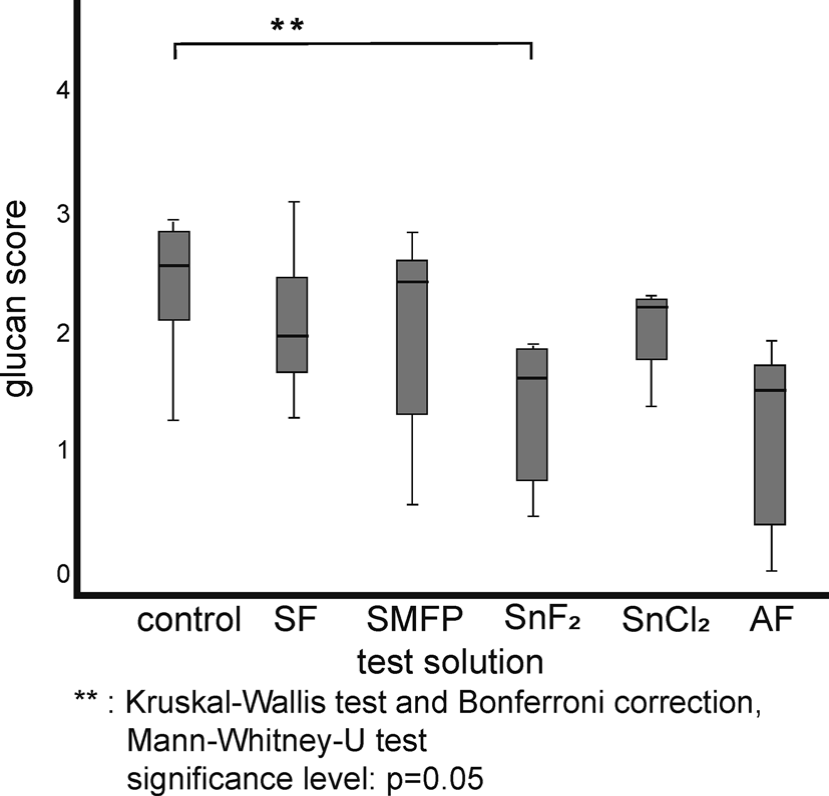
Quantification of bacterial glucan agglomerates using ConA staining illustrated in a box plot diagram. The count of glucan rings was measured after 8 h of biofilm formation and 1-min rinsing with the monosubstance test solutions at pH 4.5. Only the SnF_2_ rinsing shows a significant distinction from the control, as determined by Kruskal–Wallis and Mann–Whitney-U tests.

**Figure 8:**
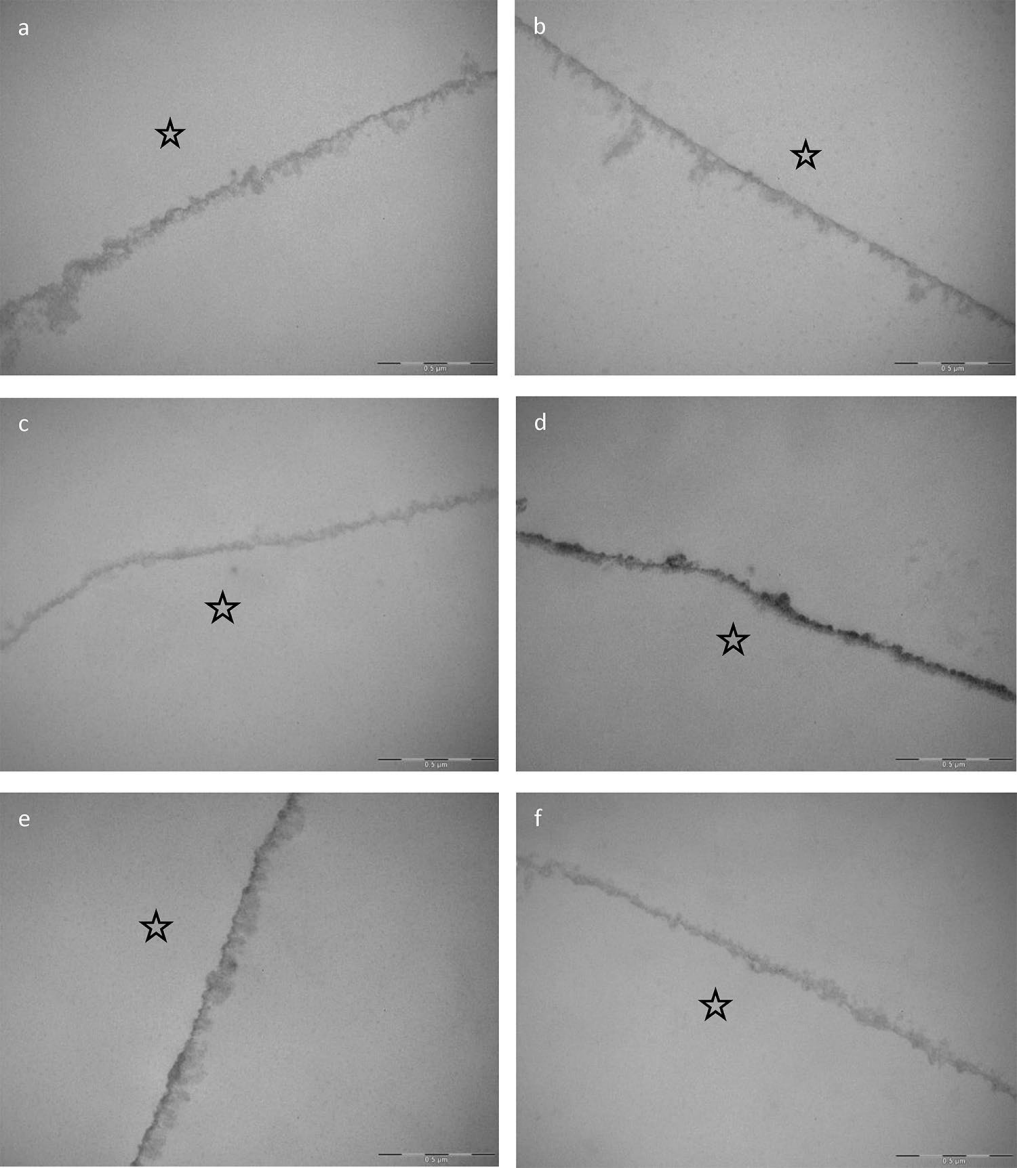
Representative TEM images of the 8-h in situ pellicle after 1 min rinsing with SF (**b**), SMFP (**c**), SnF_2_ (**d**), SnCl_2_ (**e**), and AF (**f**). The control is shown in image (**a**). The asterisk indicates the former enamel site, which was degraded during specimens processing. The pellicles show an electron dense basal layer covered by thicker outer globular pellicle structures. Control (**a**) and SF (**b**) pellicles display a pronounced external layer containing larger globular formations. The diameters of the SMFP (**c**) and AF (**f**) pellicles are comparatively small, but the pellicles still show their typical structure with an electron dense basal layer and a globular outer layer. After application of SnF_2_ (**d**) and SnCl_2_ (**e**), the basal pellicle appears more electron dense with inhomogeneous black stannous precipitations.

Figure 3 depicts representative image sections of the 8 h biofilm after staining with DAPI combined with the glucan stain ConA, while Fig. 4 shows bacterial adherence visualized with BacLight™. Quantification is shown in the box plots in Figs. 5, 6, 7 In the control group, all staining methods consistently indicated that approximately 50% of the enamel surface was predominantly colonized by coccoid bacteria (Figs. 3a and 4a). Significant differences in bacterial counts were observed between the different rinsing solutions. Rinsing with SF or SMFP did not result in a significant reduction in bacterial counts (Figs. 5b,c and 6b,c). However, rinsing with AF, SnF_2_ or SnCl_2_ significantly reduced bacterial adherence compared to the control, as observed by both DAPI and BacLight™ staining (Figs. 5 and 6 AF, SnF_2_ and SnCl_2_ with BacLight™: *p* < 0.03; AF and SnF_2_ with DAPI: *p* < 0.03; SnCl_2_ with DAPI: *p* < 0.05). AF, SnF_2_ and SnCl_2_ even yielded a significant improvement compared to a 1 min rinse with SF (Figs. 7 and 8, SnF_2_ and SnCl_2_ with DAPI: *p* < 0.05; AF with DAPI: *p* < 0.03; SNF_2_, SnCl_2_ and AF with BacLight™: *p* < 0.03). In BacLight™ LIVE/DEATH staining, rinsing with SnF_2_ significantly reduced bacterial adherence of vital and non-vital bacteria even compared to rinsing with SMFP (*p* < 0.05), as shown in Fig. 6. On the enamel surface of the control samples, the ratio of vital (green) to avital (red) bacteria was quite similar (Fig. 4). After rinsing with the test solutions, a higher number of dead bacteria than vital bacteria was detected (Fig. 4). Distinct glucan structures were observed around the bacteria in the control group and after rinsing with SMFP, SF and SnCl_2_ (Fig. 3). Glucan structures are present around almost all bacteria (Fig. 3). After rinsing with AF and SnF_2_, there was a reduction in extracellular polysaccharide structures with a statistically significant difference between control and SnF_2_ (Fig. 7, *p* < 0.05).

### TEM

Visualization of the pellicle ultrastructure after 8 h and its modification by the test solutions at pH 4.5 was carried out using TEM (Fig. 8). As expected, the control pellicle consisted of an electron-dense basal layer (10 nm) and an outer, loosely arranged and irregular layer with an average diameter of about 400 nm (Fig. 8a). Rinsing for 1 min with SF (Fig. 8b) lead to the formation of a thicker outer pellicle layer with multiple globular structures. Rinsing with SMFP (Fig. 8c) and AF (Fig. 8f) did not result in any visible changes in the ultrastructure of the pellicle, either in diameter or density. Figure 8d,e show the 8 h pellicle after modification with SnF_2_ (Fig. 8d) and SnCl_2_ (Fig. 8e). The TEM images showed dark, electron dense stannous deposits within the basal pellicle layer. After rinsing with SnF_2_ and SnCl_2_ (Fig. 8d,e), the stannous deposits did not accumulate beyond the original enamel surface as the pH 4.5 solutions themselves did not cause demineralizations. The formation of the so-called "subsurface pellicle" was not observed in our study. The basal pellicle layer appeared more electron dense after application of SnF_2_ (Fig. 8d) and SnCl_2_ (Fig. 8e) than in the control (Fig. 8a) or after rinsing with SF (Fig. 8b), SMFP (Fig. 8c) or AF (Fig. 8f). In addition, the globular outer pellicle layer was evenly thicker after rinsing with a stannous-containing test solution than after rinsing with SF (Fig. 8b), SMFP (Fig. 8c) or AF (Fig. 8f). In particular, the fluoride-free SnCl_2_ solution (Fig. 8e) produced a thick outer pellicle layer with a diameter of up to 700 nm (Fig. 8e).

## Discussion

This is the first study to investigate the influence of the pH of fluoride and stannous monosubstances on intraoral bioadhesion processes under in situ conditions. It is noteworthy that both erosion protection and the influence on bacterial colonisation as well as the modification of the pellicle ultrastructure were investigated. This allowed us to investigate whether the acidic environment or the corresponding cation is responsible for the efficacy of a rinsing solution. This has important clinical implications for the recommendation of fluoride compounds in mouthrinses or their pH adjustment to increase efficacy.

The in vitro preliminary test suggested that the monosubstances at the more acidic pH 4.5 reduce the acid-induced calcium and phosphate release more than the solutions at pH 5.5. The erosion protection effect of fluoride and/or stannous solutions could therefore be enhanced in an acidic environment. These results may be attributed to the superior erosion protection of an acidic solution, as it provides more calcium ions for the formation of a CaF_2_-layer at a low pH value^36,37^. The inferior erosion protection of the stannous containing solutions at pH 5.5 may also be due to their reduced stability in a neutral environment^38^. The considerably better effect of SnCl_2_ without pH adjustment confirms this assumption, as our SnCl_2_ solution was raised from pH 3.5 to 4.5 and may have lost stability and thus effect^24^.

In contrast to previous studies, the pH values of the fluoride and/or stannous containing solutions were uniformly adjusted in the present in vitro and in situ studies. Without adjustment, AF, SnF_2_, and SnCl_2_ have an acidic pH at a fluoride concentration of 500 ppm (AF: pH 3.6; SnF_2_: pH 4.5; SnCl_2_: pH 3.5). SF with a pH of 7.1, and SMFP with a pH of 6.6 are naturally in the neutral pH range. In two studies by our working groups, identical fluoride and stannous monosubstances were tested in situ at equivalent concentrations without pH adjustment^24,26^. These studies showed that the erosion protection provided by SnCl_2_, at its pH of 3.6, was as remarkable as that provided by SnF_2_ at a pH of 4.5^24^. The weakening of the erosion-preventive properties of SnCl_2_ observed in our study, despite maintaining the same stannous concentration of 1563 ppm, can be attributed to the use of a higher pH of 4.5. The initial dissolution of calcium ions from the enamel surface is facilitated by an acidic rinsing solution, potentially increasing the availability of calcium ions for the formation of a protective CaF_2_-like layer^39–41^. As a result, this layer would be more stable and therefore more resistant against subsequent acid attacks^39–41^. Our rinsing solutions, maintained at a constant pH of 4.5, showed equivalent antimicrobial properties to the test solutions used by Kirsch et al.^26^ without pH adjustment. At a pH of 4.5, SnCl_2_ and AF maintain their anti-cariogenic properties and SnCl_2_ still effectively prevents demineralization. Thus, a pH of 4.5 could be considered as the optimum pH in terms of solution-induced erosion prevention and anti-adherent properties.

Regarding erosion protection, the single 1 min rinse with SnF_2_ provides better protection than rinsing with SF, SMFP or AF. SnCl_2_ also reduced erosive mineral loss more effective than SF, SMFP or AF. This study is in line with the results of Kensche et al.^24^ and reinforces the effectiveness of the stannous ions containing agents. Stannous ions have a high electronegativity, which provides an excellent erosion-protective effect. They quickly form salts with dissolved calcium, phosphate, or fluoride ions, which then precipitate on the tooth surface^42–44^. This process slows down the diffusion of acid protons to the tooth surface^42^. In an acidic environment, tin ions may be incorporated into demineralised areas of the enamel, strengthening the hydroxyapatite grid^12^.

However, it remains to be emphasised that basically all fluoride-containing substances used in the study caused a distinct reduction in mineral loss compared to the control pellicle across all pH values (Fig. S1 and 3). Still, the observed reductions were not statistically significant at the low fluoride concentration of 500 ppm and with a single 1 min application. Despite the relatively modest fluoride concentration of 500 ppm, our study proves that only SnF_2_ consistently and significantly confers substantial protection against erosive effects. This protection is essentially due to the affinity of stannous ions to the hydroxyapatite matrix^45^. The presence of stannous deposits in the basal pellicle layer is demonstrated by the present TEM images (Fig. 8). Previous Energy Dispersive X-Ray (EDX) analyses consistent with our experimental protocol have already confirmed the precipitation of stannous ions within the electron-dense basal layer^26^. In addition, Kirsch et al. have elucidated the penetration of stannous ions into surface-dissolved enamel regions, characterizing this phenomenon as the ‘subsurface pellicle’. Both SnF_2_ and SnCl_2_ monosubstances have an inherently acidic environment (SnF_2_: pH 4.5, SnCl_2_: pH 3.5) at the concentrations indicated (500 ppm fluoride concentration, 1563 ppm stannous concentration). This initial acidic environment initiates the demineralization cascade at the enamel surface, where the resulting lacunae are subsequently occupied by stannous ions and pellicle proteins, leading to the formation of a so-called subsurface pellicle^26,46^. However, our experimental results do not confirm the presence of the subsurface pellicle under the moderately acidic conditions of pH 4.5 of our stannous solutions, as already suggested by Kirsch et al.^26^. In contrast, our TEM images demonstrate the presence of black stannous precipitates within the basal layer of the pellicles (Fig. 8). As SnCl_2_ is less acidic at pH 4.5 compared to its unmodified state at pH 3.5, it leads to reduced dissolution of calcium and phosphate ions from the hydroxyapatite, yet still provides effective protection against erosive demineralization.

In addition to serving as an acid protective barrier, the pellicle must also be considered as the initial site of bacterial colonization. If fluoride and/or stannous solutions have an impact on the acquired enamel pellicle, then it also affects the dental biofilm. The antimicrobial properties of various fluorides and stannous solutions have been extensively studied^10,13,26,47–49^. These properties are long-lasting, with stannous ions showing high substantivity on dental hard tissues^45^. In addition to increasing pellicle formation on stannous-containing surfaces due to the double positive charge of stannous ions, they also inhibit the glycolysis enzymes aldolase and glyceraldehyde-3-phosphate-dehydrogenase, which are necessary for bacterial metabolism^50^. Our study also demonstrated the antimicrobial properties of test solutions containing organic fluoride and/or stannous ions. AF, SnF_2_, and SnCl_2_ significantly reduced bacterial counts compared to the control group without rinsing solution and even compared to the SF test solution (DAPI, Fig. 5). Using the BacLight™ LIVE/DEAD staining method, the number of vital and dead bacteria was significantly lower after rinsing with SnF_2_ compared to rinsing with SF or SMFP (Fig. 6). AF rinsing also resulted in a significant reduction of vital bacteria compared to SF rinsing (Fig. 6), which is also confirmed by Kirsch et al.^26^, although they used an AF rinsing without pH adjustment. This shows that AF maintains its anti-adherent efficacy despite the change in pH from 3.6 to 4.5^26^. The remarkable antimicrobial properties of AF and stannous solutions may be related to the positive charge of AF and the influence of stannous ions on the pellicle proteome. This subject has been extensively explored by other authors^47,51,52^. The positively charged amino group of AF together with the doubly positively charged stannous ions, blocks calcium binding sites on hydroxyapatite under acidic conditions, limiting bacterial adhesion to tooth surfaces^51^. Rinsing with stannous fluoride also affects the pellicle proteome^14,47^. Algarni et al.^47^ found significantly increased protein content in the in vitro pellicle after combined rinsing with SnCl_2_ and SF, with higher levels of mucins, albumin, and carbonic anhydrase. They speculated that this observation could be due to the double positive charge of stannous ions, which have a higher affinity for salivary proteins compared to the single positively charged sodium ions^47^. In addition, stannous ions and AF may affect bacterial metabolism^48–50,53^. Stannous ions oxidize thiol groups of bacterial enzymes directly inhibiting bacterial adhesion^11,52^. They exert antibacterial effects by inhibiting enzymes involved in glucose transport and metabolism in bacterial cells^11,52^. The effect of stannous ions on bacterial metabolism also extends to their involvement in gene expression^49,54^. Gumber et al.^49^ compared the gene pathways of 48-h in situ biofilms after application of SnF_2_ and SF toothpaste. They found that fewer genes responsible for bacterial metabolism, cell wall growth, and overall bacterial growth were expressed after SnF_2_ application. Increased expression of MraZ, a negative transcriptional regulator of cell wall division, was detected^49^. Thus, stannous ions inhibit bacterial cell wall division and consequently their growth^49^. The significantly lower glucan score observed in our study (Figs. 3 and 7) after rinsing with SnF_2_ can also be attributed to the influence of stannous ions on gene expression, as Gumber et al.^49^ discovered the inhibition of bacterial pectinase expression, an enzyme in glucan metabolism, after the application of SnF_2_.

In the present study, in addition to the influence of pH, we observed that the rinsing solutions provided greater protection against erosive tissue loss when used in situ then used in vitro. For in vitro pellicle formation, freshly collected saliva from 1 participant was used. The natural pellicle contains not only salivary components but also proteins from the sulcular fluid and cellular elements from the mucosal epithelium^55,56^. In vivo, the pellicle undergoes considerable enzymatic remodelling and continuous maturation^56^. Epithelial cells and components of the oral sulcular fluid cannot be incorporated into the pellicle in vitro. It can be concluded, that only in situ experiments are suitable to adequately investigate pellicle formation influenced by rinsing solutions. The complex processes of pellicle formation and maturation are difficult to replicate in vitro, meaning that in vitro experiments are only suitable for preliminary testing.

In our studies, we have not noticed any negative side effects from rinsing with stannous-containing solutions, such as astringent sensations or a dull feeling on the enamel surface. Tooth staining, which could also be a negative side effect of stannous containing solutions, was not tested in our studies because there were no clinical examinations after the use of the solutions. In contrast, previously published studies have reported some side effects following the use of highly concentrated stannous fluoride solutions or other polyvalent metal cation solutions^44,57^. We probably did not find any negative side effects due to the low stannous concentration of our monosubstances and the single application of the test solution.

The erosion-preventing properties of SF, SMFP, AF, and SnCl_2_ at 500 ppm fluoride concentration were not found to be significant after a single application. Kensche et al.^24^ also investigated various fluoride or stannous monosubstances at a fluoride concentration of 500 ppm. With the stannous-free test solutions, they were equally unable to demonstrate any significant erosion protection. For future research, it would be interesting to investigate application over several days in accordance with a daily prophylaxis regimen. Marinho et al.^2^ were able to prove that regular use of fluoride mouthrinses reduces the progression of biofilm-induced caries, even at low fluoride concentrations of 230 or 900 ppm. Finally, Fluorides are an essential component in home prevention of erosion and biofilm induced diseases such as caries.

## Conclusions

The present study demonstrated an erosion protection provided by fluoride and/or stannous solutions influenced by the pH value. Thereby, the influence of the pH of the respective solutions on their anti-adhesive effect is of minor importance. Fluoride cations have the strongest influence on erosion inhibition and bacterial adhesion reduction. However, only stannous ions provide sufficient erosion protection after a single application.

Rinses with AF and SnCl_2_ monosubstances, a pH of 4.5 is ideal to maintain a balance between erosion protection and biocompatibility.

### Supplementary Information

Supplementary Figure S1.

Supplementary Figure S2.

Supplementary Figure S3.

Supplementary Figure S4.

Supplementary Figure S5.

Supplementary Figure S6.

Supplementary Figure S7.

Supplementary Figure S8.

Supplementary Information 9.

## Data Availability

All data generated or analysed during this study are included in this published article (and its Supplementary Information files).
