# Supplementary figures and images for: Impact of pH-adjusted fluoride and stannous solutions on the protective properties on the pellicle layer in vitro and in situ
Source: Sci Rep. 2024 Feb 9;14:3378. doi: 10.1038/s41598-024-53732-7 (PMC10858267; doi:10.1038/s41598-024-53732-7)

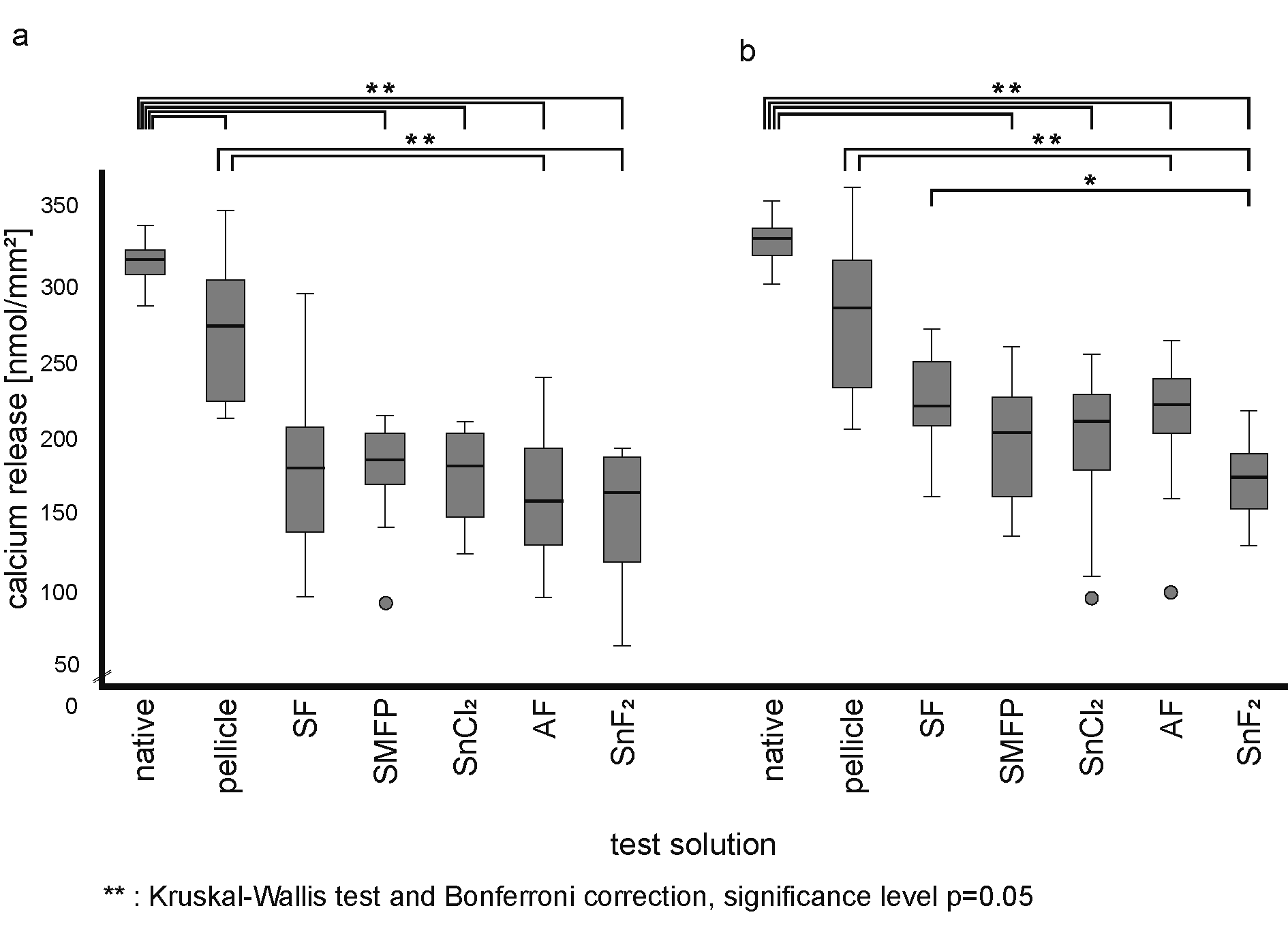

Supplement: Supplementary file 1 — Supplementary Figure S1. [file 41598_2024_53732_MOESM1_ESM.tif]

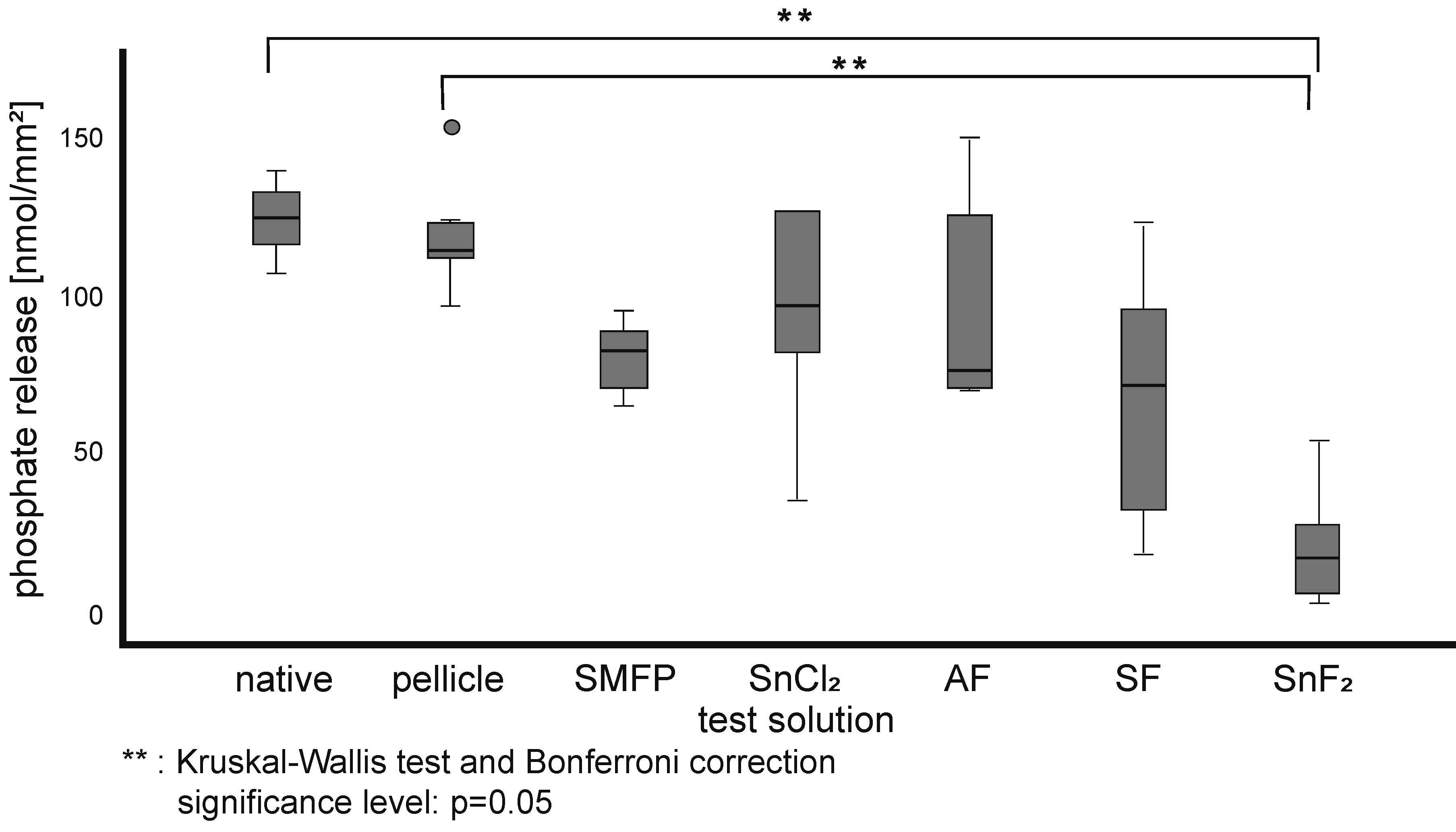

Supplement: Supplementary file 2 — Supplementary Figure S2. [file 41598_2024_53732_MOESM2_ESM.tif]

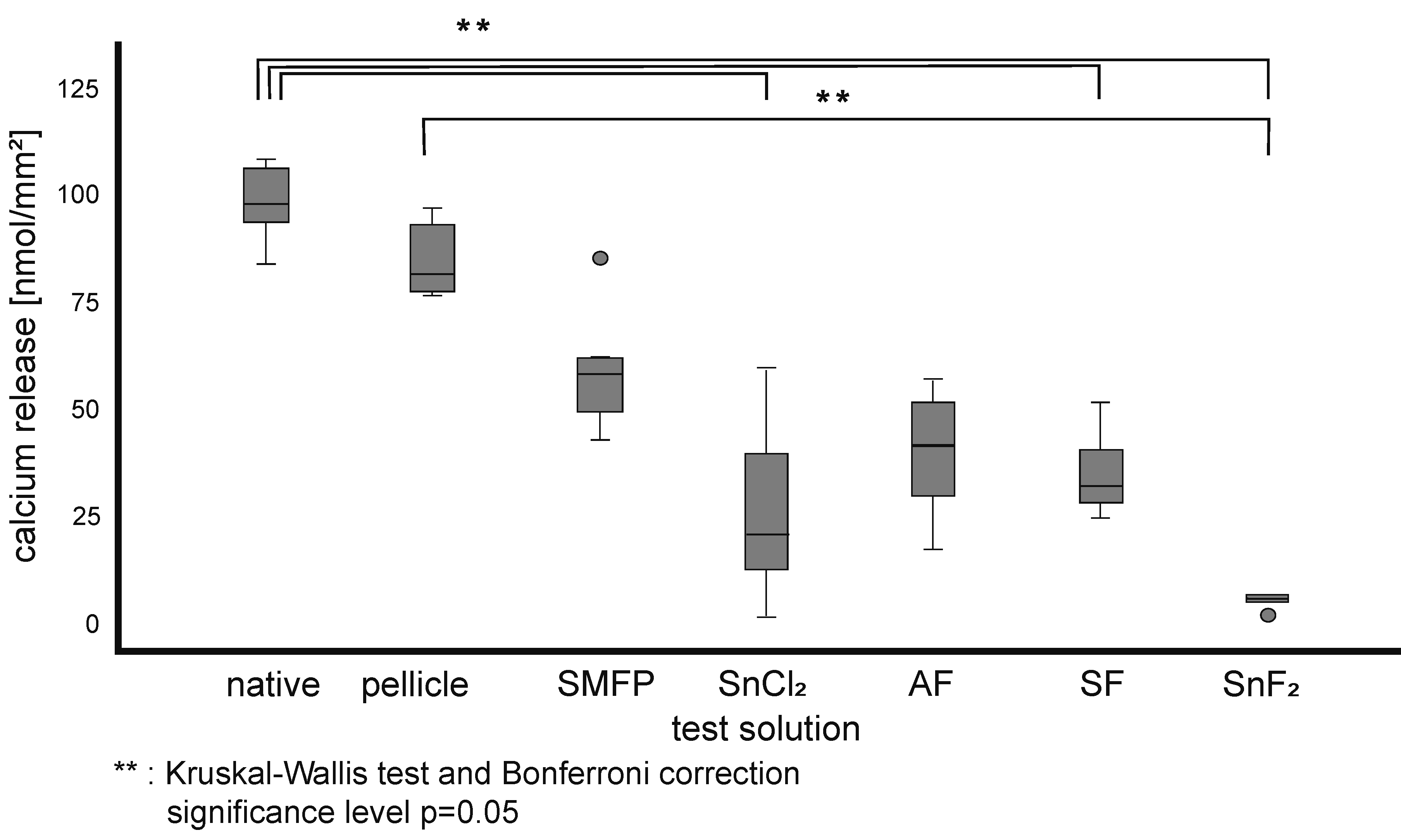

Supplement: Supplementary file 3 — Supplementary Figure S3. [file 41598_2024_53732_MOESM3_ESM.tif]

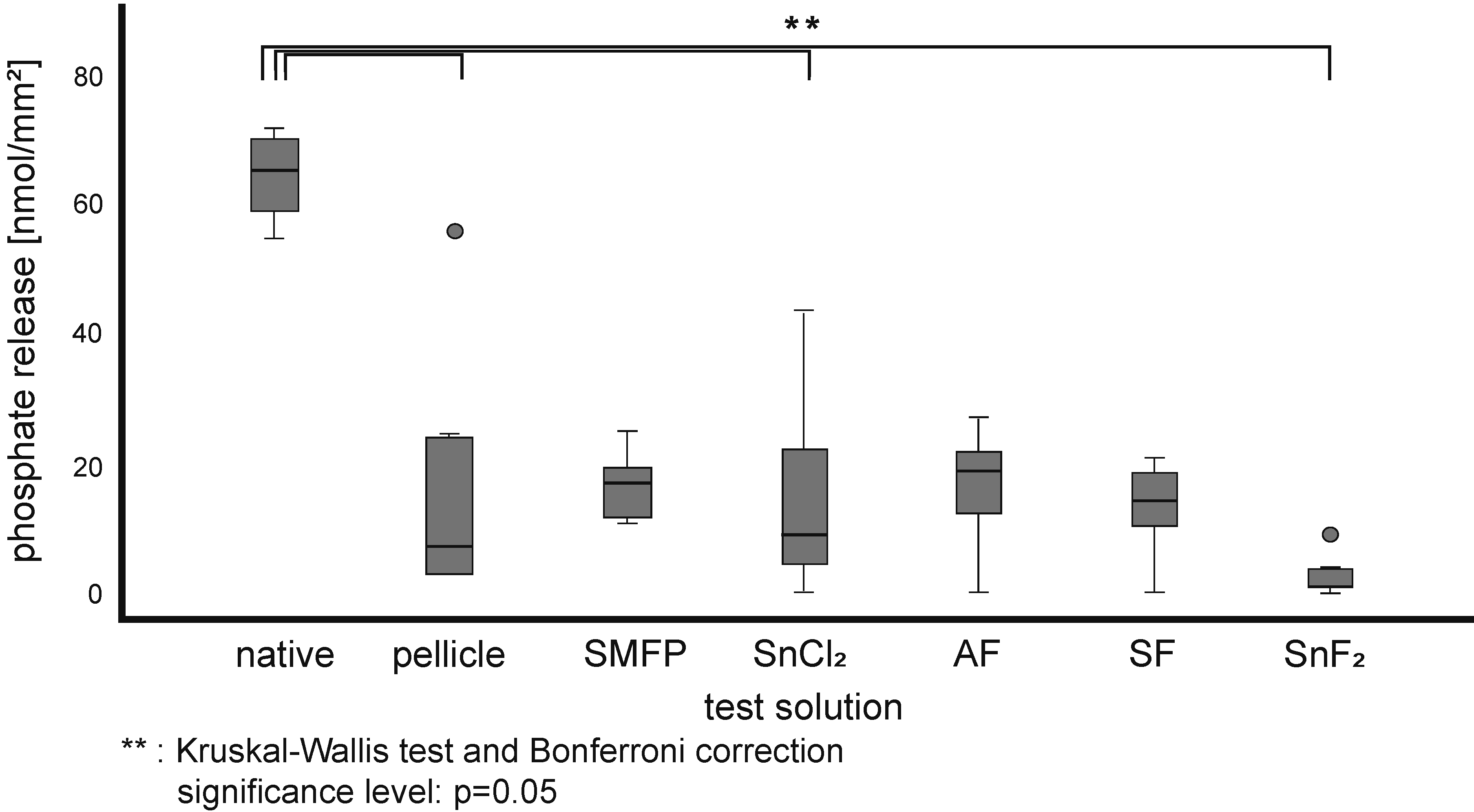

Supplement: Supplementary file 4 — Supplementary Figure S4. [file 41598_2024_53732_MOESM4_ESM.tif]

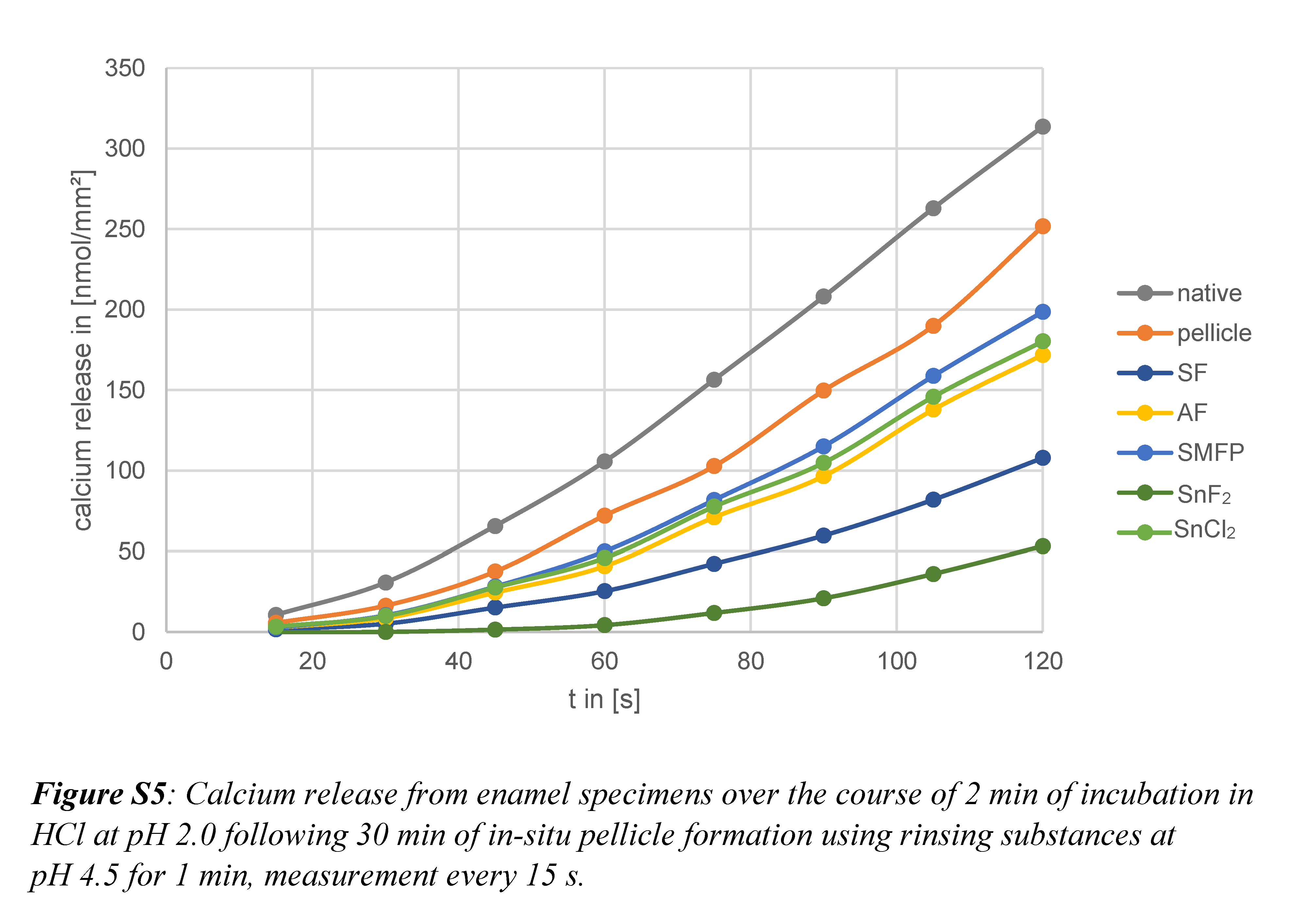

Supplement: Supplementary file 5 — Supplementary Figure S5. [file 41598_2024_53732_MOESM5_ESM.tif]

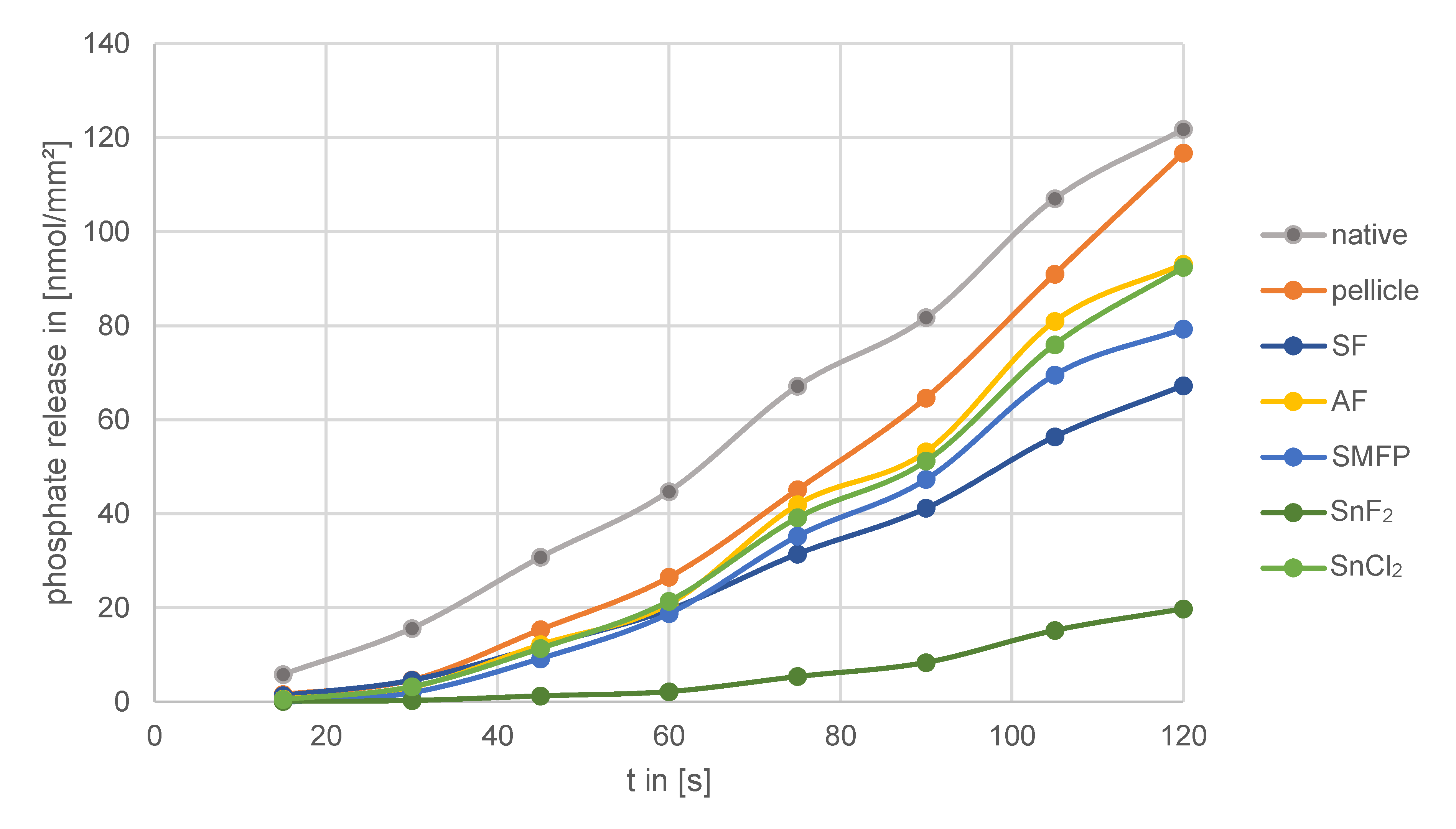

Supplement: Supplementary file 6 — Supplementary Figure S6. [file 41598_2024_53732_MOESM6_ESM.tif]

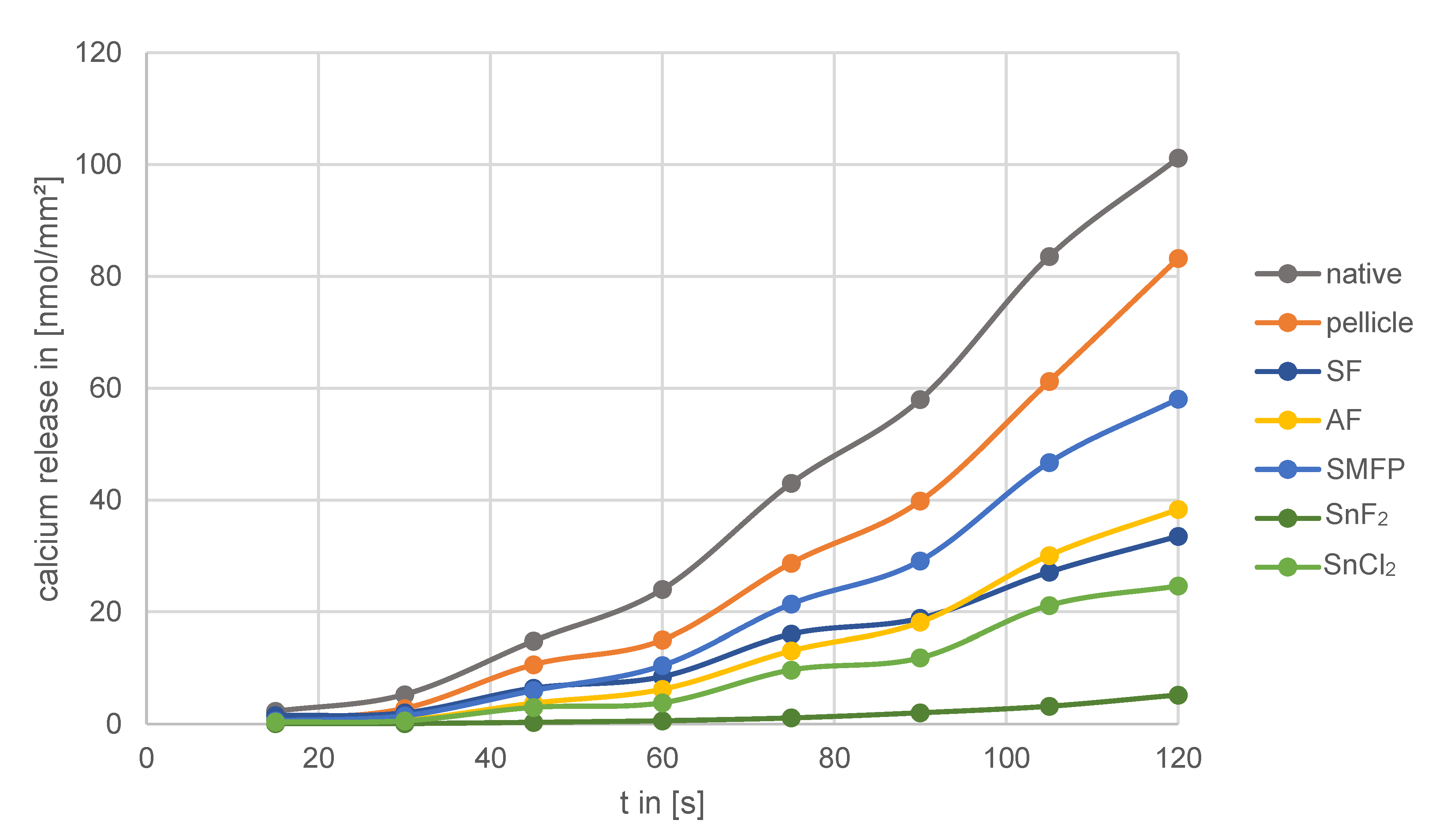

Supplement: Supplementary file 7 — Supplementary Figure S7. [file 41598_2024_53732_MOESM7_ESM.tif]

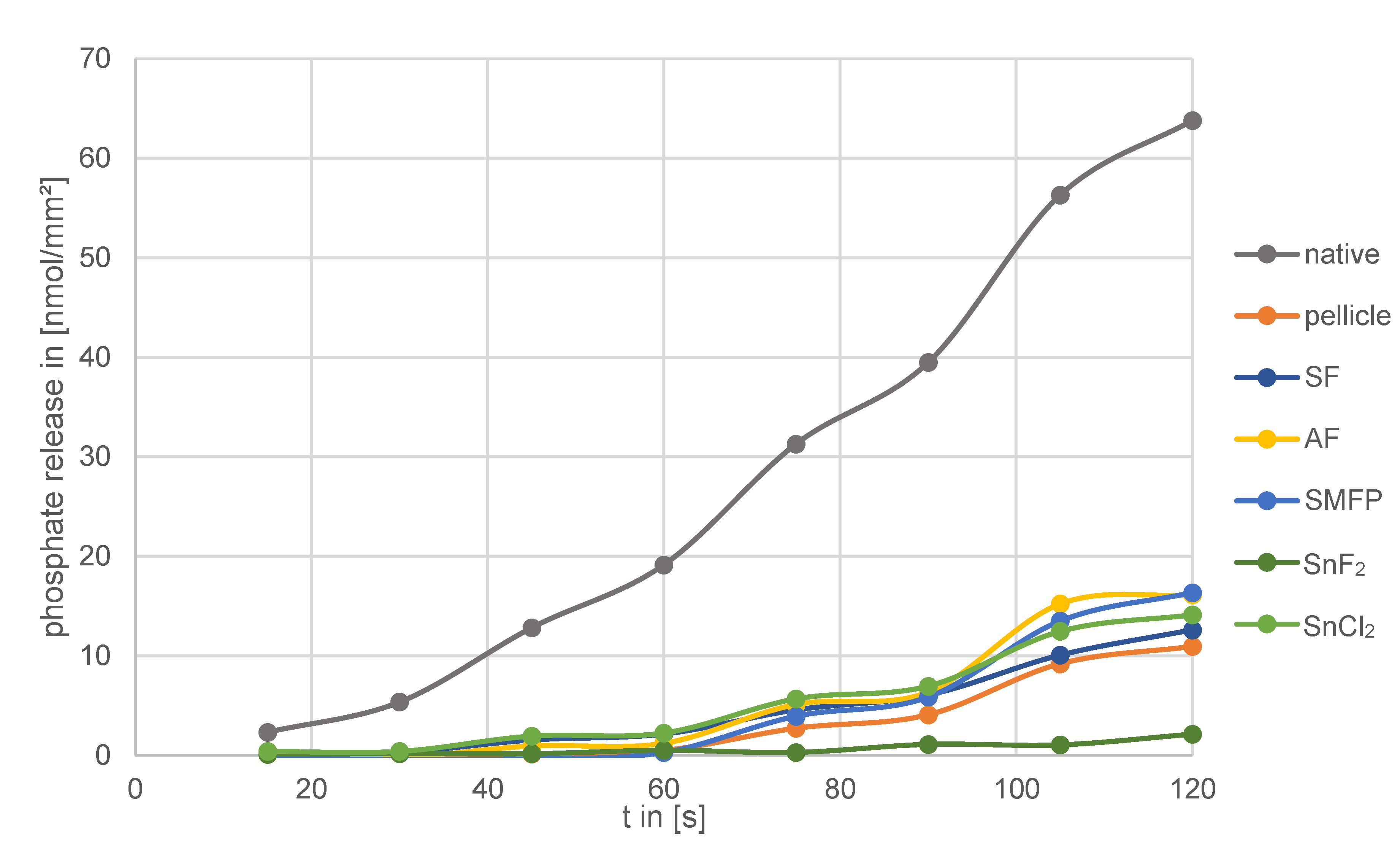

Supplement: Supplementary file 8 — Supplementary Figure S8. [file 41598_2024_53732_MOESM8_ESM.tif]
