# Supplementary material for: Impact of pH-adjusted fluoride and stannous solutions on the protective properties on the pellicle layer in vitro and in situ
Source: Sci Rep. 2024 Feb 9;14:3378. doi: 10.1038/s41598-024-53732-7 (PMC10858267; doi:10.1038/s41598-024-53732-7)
Supplement: Supplementary file 9 — Supplementary Information 9. [file 41598_2024_53732_MOESM9_ESM.docx]

**Methods**

*Pellicle formation in vitro*

The experimental setup for *in‑vitro* and *in‑situ* pellicle formation is shown in figure S1. Altogether 314 enamel samples were used for the *in-vitro* studies. The number of slabs is based on the *in-vitro* study sample calculation performed for each test solution prior to the start of the study. For *in vitro* pellicle formation, the samples were incubated in freshly collected saliva at 37°C, which ensured similar conditions to those for in situ pellicle formation. The samples were agitated gently for 60 min. The saliva donor (healthy, caries-free non-smoker with a physiological salivary flow) did not consume anything except water for up to 2 h before the start of the tests. After 30 min, the saliva was replaced with the test solution at pH of 4.5 or 5.5 for 60 s and incubated in saliva for further 29 minutes. As an *in‑vitro* control group, test specimens were incubated in saliva for 60 min without any rinsing. The specimens were stored at 4 C° for a maximum of 30 min until analysis.

*Calcium- and phosphate release in vitro*

Figure S1 depicts the calcium release from enamel slabs after 120 s of incubation in HCl at pH 2.0, including statistical analysis. All fluoride and/or stannous containing test solutions yield a substantial reduction in calcium and phosphate release compared to the control, consisting of native enamel and a pellicle layer. The 60 min *in‑vitro* pellicle provides an insufficient and statistically non-significant level of erosion protection. Specifically, SnF_2_ shows a significant improvement in erosion protection compared to both control conditions (reduction of calcium release: - 47 % at pH 2.0, p < 0.002; - 67 % at pH 2.3, p < 0.001). SnCl_2_ also enhances erosion protection of the *in‑vitro* pellicle, although it does not contain any fluoride. In addition, the *in‑vitro* pre-tests suggest that the monosubstances provide a more pronounced erosion protection effect in an acidic environment. Fluoride and/or stannous containing solutions tend to reduce calcium and phosphate release more at pH 4.5 (Fig. 2a) than at pH 5.5 (Fig. 2b), although this is not statistically significant.

**Results**





***Figure S1****: Preliminary in-vitro-experiments: Calcium release from enamel specimens after 120 s of incubation in HCl at pH 2.0 following 60 min of in‑vitro pellicle formation using rinsing solutions at pH 4.5 (a) and 5.5 (b) for 1 min. Statistical analysis performed using the Kruskal-Wallis test and Bonferroni correction (p < 0.05).*


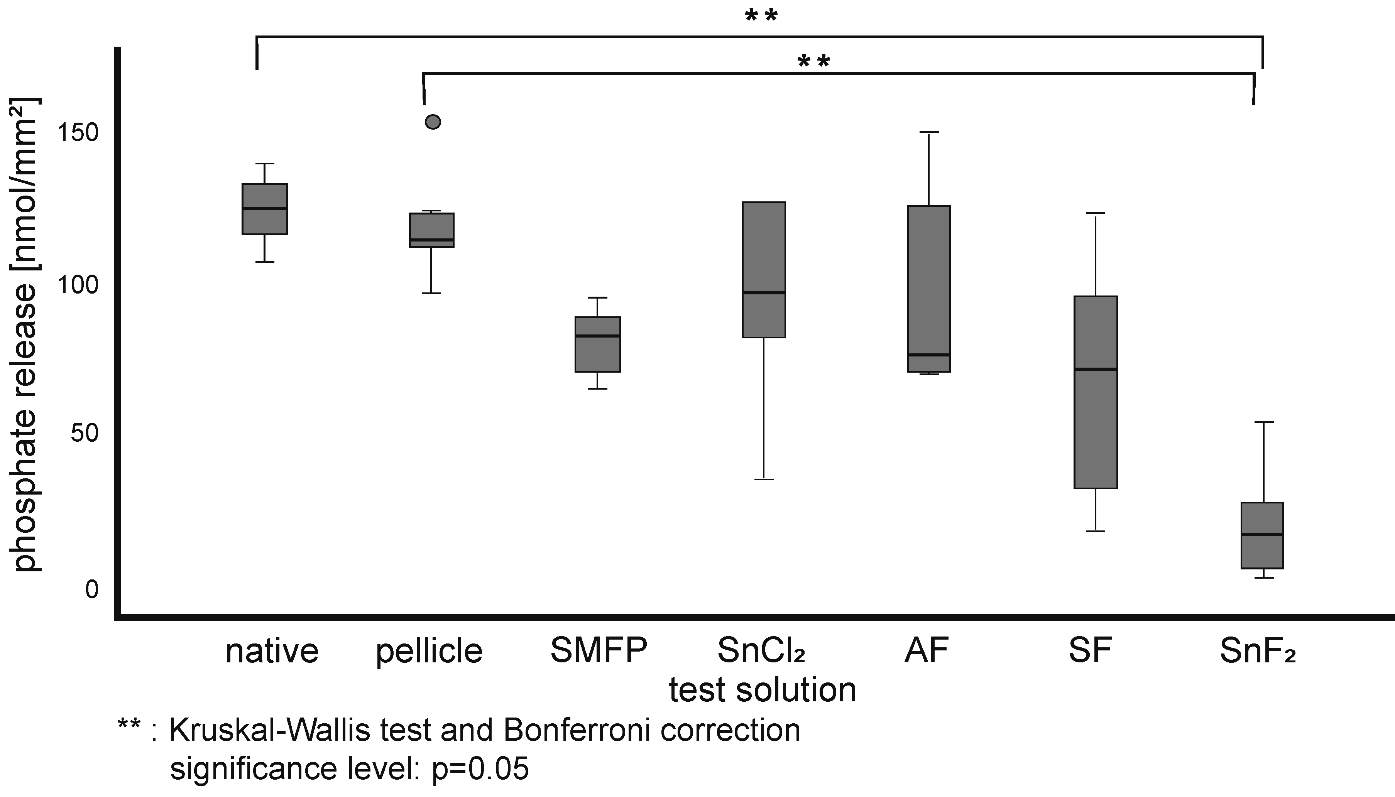


***Figure S2:*** *Phosphate release from enamel specimens after 120 s of incubation in HCl at pH 2.0 following 30 min of in‑situ pellicle formation using rinsing substances at pH 4.5 for 1 min. Statistical analysis performed using the Kruskal-Wallis test and Bonferroni correction (p < 0.05).*

*
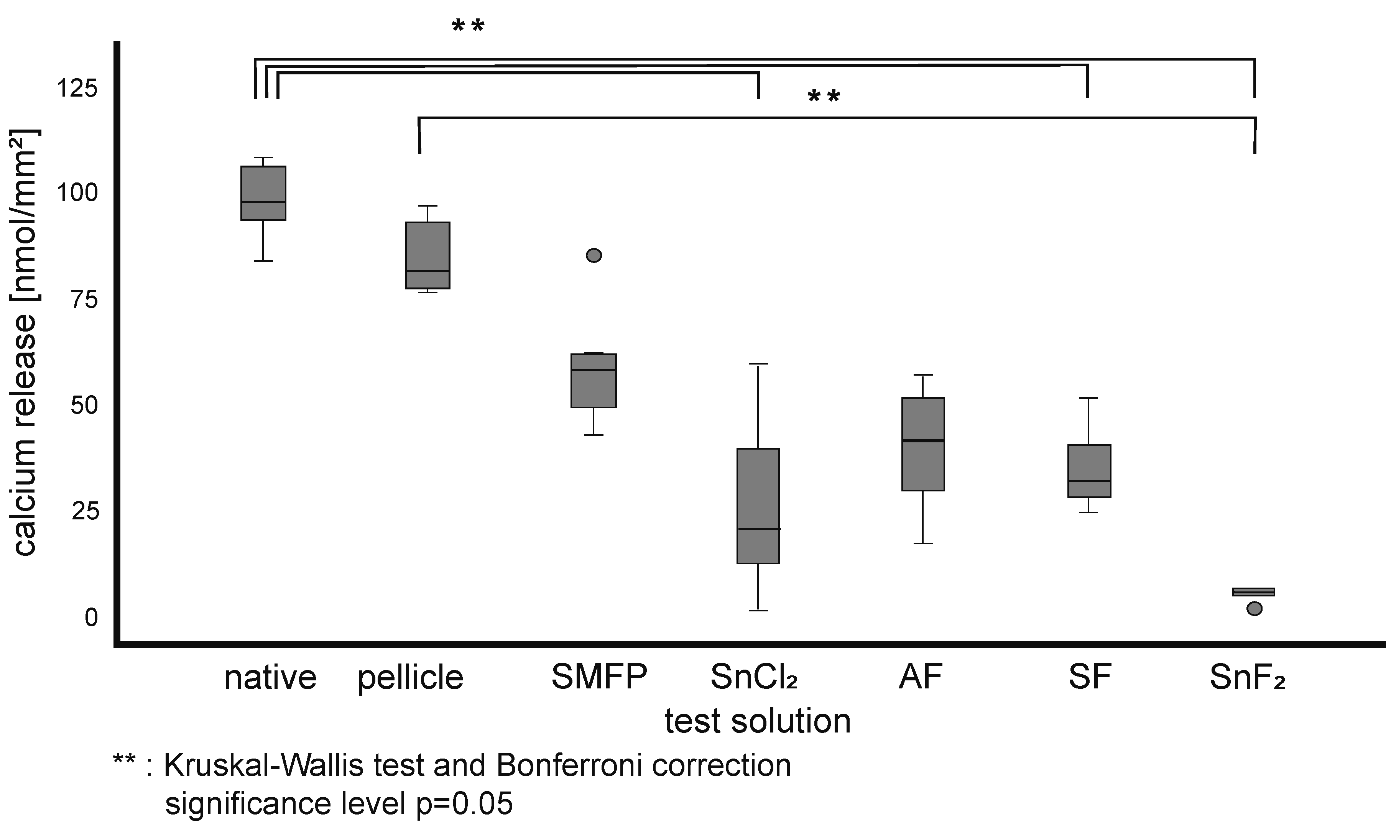
*

***Figure S3:*** *Calcium release from enamel specimens after 120 s of incubation in HCl at pH 2.3 following 30 min of in‑situ pellicle formation using rinsing substances at pH 4.5 for 1 min. Statistical analysis performed using the Kruskal-Wallis test and Bonferroni correction (p < 0.05).*

***
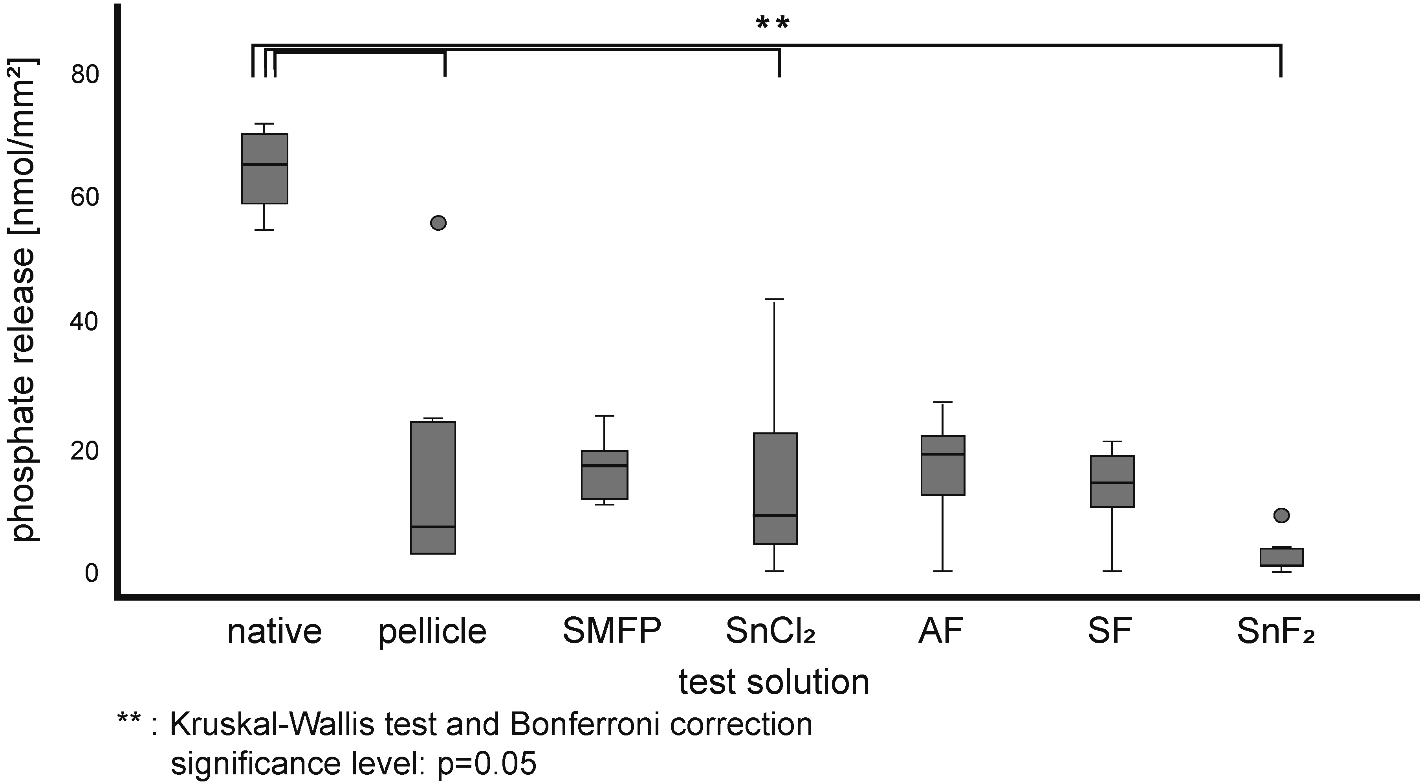
***

***Figure S4****: Phosphate release from enamel specimens after 120 s of incubation in HCl at pH 2.3 following 30 min of in‑situ pellicle formation using rinsing substances at pH 4.5 for 1 min. Statistical analysis performed using the Kruskal-Wallis test and Bonferroni correction (p < 0.05).*

SnF_2_

SnCl_2_

SnCl_2_

***Figure S5****: Calcium release from enamel specimens over the course of 2 min of incubation in HCl at pH 2.0 following 30 min of in-situ pellicle formation using rinsing substances at pH 4.5 for 1 min, measurement every 15 s.*

SnF_2_

SnCl_2_

***Figure S6****: Phosphate release from enamel specimens over the course of 2 min of incubation in HCl at pH 2.0 following 30 min of in-situ pellicle formation using rinsing substances at pH 4.5 for 1 min, measurement every 15 s.*

SnCl_2_

SnF_2_

***Figure S7****: Calcium release from enamel specimens over the course of 2 min of incubation in HCl at pH 2.3 following 30 min of in-situ pellicle formation using rinsing substances at pH 4.5 for 1 min, measurement every 15 s.*

SnF_2_

SnCl_2_

***Figure S8****: Phosphate release from enamel specimens over the course of 2 min of incubation in HCl at pH 2.3 following 30 min of in-situ pellicle formation using rinsing substances at pH 4.5 for 1 min, measurement every 15 s.*
